# Whole-Genome Sequence Approach and Phylogenomic Stratification Improve the Association Analysis of Mutations With Patient Data in Influenza Surveillance

**DOI:** 10.3389/fmicb.2022.809887

**Published:** 2022-04-19

**Authors:** Laura Van Poelvoorde, Kevin Vanneste, Sigrid C. J. De Keersmaecker, Isabelle Thomas, Nina Van Goethem, Steven Van Gucht, Xavier Saelens, Nancy H. C. Roosens

**Affiliations:** ^1^Transversal Activities in Applied Genomics, Sciensano, Brussels, Belgium; ^2^National Influenza Centre, Sciensano, Brussels, Belgium; ^3^Department of Biochemistry and Microbiology, Ghent University, Ghent, Belgium; ^4^VIB-UGent Center for Medical Biotechnology, VIB, Ghent, Belgium; ^5^Public Health and Genome, Epidemiology and Public Health, Sciensano, Brussels, Belgium

**Keywords:** influenza, surveillance, mutations, Nextstrain, next-generation sequencing

## Abstract

Each year, seasonal influenza results in high mortality and morbidity. The current classification of circulating influenza viruses is mainly focused on the hemagglutinin gene. Whole-genome sequencing (WGS) enables tracking mutations across all influenza segments allowing a better understanding of the epidemiological effects of intra- and inter-seasonal evolutionary dynamics, and exploring potential associations between mutations across the viral genome and patient’s clinical data. In this study, mutations were identified in 253 Influenza A (H3N2) clinical isolates from the 2016-2017 influenza season in Belgium. As a proof of concept, available patient data were integrated with this genomic data, resulting in statistically significant associations that could be relevant to improve the vaccine and clinical management of infected patients. Several mutations were significantly associated with the sampling period. A new approach was proposed for exploring mutational effects in highly diverse Influenza A (H3N2) strains through considering the viral genetic background by using phylogenetic classification to stratify the samples. This resulted in several mutations that were significantly associated with patients suffering from renal insufficiency. This study demonstrates the usefulness of using WGS data for tracking mutations across the complete genome and linking these to patient data, and illustrates the importance of accounting for the viral genetic background in association studies. A limitation of this association study, especially when analyzing stratified groups, relates to the number of samples, especially in the context of national surveillance of small countries. Therefore, we investigated if international databases like GISAID may help to verify whether observed associations in the Belgium A (H3N2) samples, could be extrapolated to a global level. This work highlights the need to construct international databases with both information of viral genome sequences and patient data.

## Introduction

Influenza A virus displays the highest diversity of all influenza viruses and remains a major public health threat in developed as well as in developing countries (Gatherer, 2009). Although influenza infections are mostly mild (Allen and Ross, 2018), some population strata are at high risk for developing complications (Centers for Disease Control and Prevention [CDC], 2019). There are currently mainly two influenza A subtypes circulating in humans, namely A (H1N1) pdm09 and A (H3N2) (WHO, 2019). In particular, subtype A (H3N2) has led to numerous seasonal epidemics and is considered to evolve faster than other subtypes (Bedford et al., 2014). In recent years, A (H3N2) has shown extensive clade diversity and increased morbidity and mortality, especially in the elderly (Simon et al., 2019). This rapid evolution is mainly caused by constantly occurring mutations and intra-subtype reassortment, resulting in low vaccine effectiveness through mismatches between the vaccine strain and circulating influenza strains.

Currently, the “World Health Organization” (WHO) and “European Centre for Disease Prevention and Control” (ECDC) still focus on the genetic surveillance of the HA segment (European Centre for Disease Prevention and Control, 2014). In the context of influenza surveillance and vaccine strain selection different clades and subclades within each influenza subtype are defined based on its phylogenetic analysis and amino acid differences (European Centre for Disease Prevention and Control, 2018). However, as next-generation sequencing (NGS) has become more widely accessible, whole-genome sequencing (WGS) and obtaining sequences from all eight influenza segments simultaneously becomes cost-efficient (Zhou et al., 2009). WGS data can be used for several purposes including to improve the influenza surveillance, when appropriate approaches and analysis are applied on a dataset. To illustrate these approaches, we have previously sequenced influenza samples collected in the context of the surveillance of the 2016-2017 influenza season in Belgium. In a first study, this dataset was used to demonstrate that using powerful phylogenomic tools such as BEAST and Nextstrain, allows substantially improved phylogenetic classification when considering the whole genome rather than solely the HA segment (Van Poelvoorde et al., 2021). Furthermore, Bayesian inference via BEAST allowed reassortment detection by both computational methods and manual inspection. These combined methods resulted in an estimated rate of 15% intra-subtype reassortment for A (H3N2) samples from the Belgian 2016-2017 outbreak season. Additionally, A (H3N2) reassortants were found to be more likely to infect hospitalized patients compared to patients with mild symptoms, which would not have been possible without considering the whole genome (Van Poelvoorde et al., 2021). In another study, we have used genomic data from the hospitalized patients in this dataset in a predictive model and assessed the added value of viral genomic data in addition to clinical information (Van Goethem et al., 2021). The aim of the current study is to evaluate whether whole genome information may also enable exploring mutations located on all eight segments. Moreover, by integrating with patient data, associations of viral mutations and patients’ characteristics can be detected, including the disease severity. In contrast to bacterial infections, clinical studies exploring the link between mutations and the disease severity remain scarce (Wedde et al., 2013). The pathogenesis of viruses is dependent on complex and unpredictable mechanisms, including interactions formed within and between the influenza proteins. Consequently, certain mutations cannot be considered individually, but should be considered together with mutations present in the entire genome, i.e., the genetic background (Abed et al., 2006) that evolves fast due to the highly error-prone influenza replication (Abed et al., 2006; Chandler et al., 2013). It is therefore more appropriate to include virological genetic information of all 8 segments and metadata of the host to investigate influenza (Hung et al., 2010; Lai et al., 2016). Most current studies focus on linking mutations to broadly defined patient outcomes related to vaccine efficacy or disease severity. However, assessing associations with the sampling period and additional patient information such as vaccination status, patient age, existing patient comorbidities, sex, and specific severity indicators has, to the best of our knowledge, not yet been performed.

In this study, 253 Influenza A (H3N2) whole genome sequences from the Belgian surveillance, for which phylogenetic classification has been previously reported (Van Poelvoorde et al., 2021), were used to explore potential associations between mutations positioned across the whole genome and patient characteristics and other metadata. In this analysis, the effect of sampling stratification according to the phylogenetic clade was also evaluated to consider potential effects related to the highly diverse genetic background of A (H3N2) strains. Additionally, we evaluated whether the observed associations with a restricted number of samples at the Belgian level correspond to trends observed at an international level, and highlight the necessity of constructing a large database containing both viral genome sequences and information on patient data.

## Materials and Methods

### Sample Selection, RNA Isolation, PCR Amplification, and WGS

Two sentinel surveillance systems are in place in Belgium to monitor “influenza-like-illness” (ILI) in the general practices and “severe-acute-respiratory-infections” (SARI) in the hospitals. ILI cases are defined by a sudden onset of symptoms, including fever and respiratory and systemic symptoms. A SARI case is defined as an acute respiratory illness with onset within the previous 10 days of fever, respiratory symptoms, and the requirement for hospitalization. These surveillance systems are essential for following trends of viral spread and changes in circulating influenza viruses. The present study uses 253 samples collected during the 2016-2017 influenza season in Belgium from the two surveillance systems, as previously described (Van Poelvoorde et al., 2021). These include 160 hospitalized SARI patients (mean age = 70 years) and 93 ILI outpatients (mean age = 39 years). The absence of other respiratory viruses in the sample was confirmed by RT-qPCR-based testing for respiratory syncytial virus A and B, parainfluenza viruses, enterovirus D68, rhinoviruses, human metapneumovirus, paraechoviruses, bocaviruses, adenovirus, coronaviruses OC43, NL63, 229, and MERS-CoV (Brittain-Long et al., 2008; Hombrouck et al., 2012). Samples of ILI outpatients were categorized as mild cases (*n* = 93). Samples from hospitalized SARI patients were categorized as moderate (*n* = 122) or severe cases (*n* = 38). As the requirement for hospitalization is part of the SARI case definition, all SARI cases are consequently hospitalized patients. However, hospitalization by itself was not considered as a disease severity indicator because patients could have been hospitalized for isolation purposes or due to other medical conditions. A severe case was therefore defined within the SARI population by the presence of at least one of the following severity indicators: death, stay in an intensive care unit (ICU), need for invasive respiratory support or extracorporeal membrane oxygenation (ECMO) or acute respiratory distress syndrome (ARDS). Available patient data are listed in Table 1 in conjunction with the number of patients. The nucleic acid content of the samples was extracted directly from the clinical specimens and subjected to WGS as previously described (Van Poelvoorde et al., 2021). Generated WGS data has been deposited in the NCBI Sequence Read Archive (SRA) [33] under accession number PRJNA615341. A central ethical committee and the local ethical committees of each participating hospital approved the SARI surveillance protocol (reference AK/12-02-11/4111; in 2011: Centre Hospitalier Universitaire St-Pierre, Brussels, Belgium; from 2014 onward: Universitair Ziekenhuis Brussel, Brussels, Belgium). Informed verbal consent was obtained from all participants or parents/guardians.

**TABLE 1:** Sample numbers per patient data.

Age (years):	<15	15 – 59	≥60
Beginning of epidemic (< week 4)	12	17	35
Peak of epidemic (week 4 - 6)	16	26	86
End of epidemic (> week 6)	11	16	34
Male*	122	Female*	122
Vaccinated*	52	Not vaccinated*	130
Antibiotics administered*	100	No antibiotics administered*	126
Respiratory disease*	50	No respiratory disease*	199
Cardiac disease*	54	No cardiac disease*	195
Obesity	20	No obesity	233
Renal insufficiency	35	No renal insufficiency	218
Hepatic insufficiency	6	No hepatic insufficiency	247
Diabetes	27	No Diabetes	226
Immunodeficiency	23	No immunodeficiency	230
Neuromuscular disease	21	No neuromuscular disease	232
Stay in ICU	22	No stay in ICU	231
Fatal	19	Not fatal	234

These statistics were based on a national collection containing 93 ILI (mild) samples and 160 SARI (moderate = 122; severe = 38) samples. *Samples for which certain patient data was unknown, were excluded for analyzing that particular aspect.

The genome consensus sequences were obtained as previously described (Van Poelvoorde et al., 2021), and are available in the GISAID database as isolates ID EPI_ISL_415199 to EPI_ISL_415452 [39]. Identification of genome mutations requires a closely-related reference genome. In this study, the whole genome of 2016-2017 A(H3N2) vaccine strain A/HongKong/4801/2014 (GISAID: EPI_ISL_198222) was used as a reference. This strain was used as the reference because it should be genetically close to the patient samples for that season. The obtained genome consensus sequences were aligned using ClustalW in Mega 7.0.18 with default settings. The H3 numbering, excluding the signal peptide of 16 amino acids, was used to enumerate positions (both amino acid residues and the corresponding nucleotides) in the HA protein compared to this reference strain. Samtools depth 1.3.1 (Li et al., 2009) was used to extract the coverage at each position for each sample from the BAM files. Regions with a sequencing depth lower than 100X were discarded. For two samples (A/Belgium/S0978/2017 and A/Belgium/S0182/2017), a part of the PB2, PB1, or NP fragment had a coverage lower than 100X and mutations found in these regions of these samples were consequently not considered. Additionally, mutations that occurred in less than 5% or more than 95% of all samples were also discarded as these will not contribute to the detection of associations between the mutation and the patient data.

### Phylogenomic Analysis and Subsampling by Group

**FIGURE 1:**
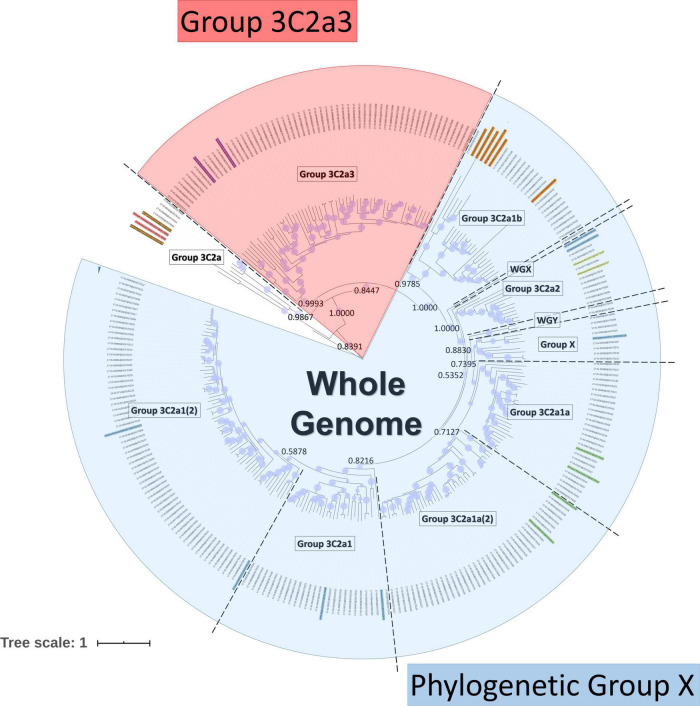
Phylogenetic tree based on the whole H3N2 genome. Within the tree, the group labels represent the phylogenetic groups that were assigned to their respective samples according to their classification based on references (colored names) and the support of nodes by posterior probability values. Posterior probability values are indicated on key nodes that separate phylogenetic groups. The size of blue disks on nodes represents the posterior probability scaled between 0.5 and 1. The scale bar represents the average number of substitutions per site. Samples belonging to ‘Phylogenetic Group X’ are indicated in blue and those belonging to Group 3C2a3 in red.

A Bayesian phylogenetic tree was created as previously described (Van Poelvoorde et al., 2021). The protein-coding sequences of the sequenced samples and references were aligned using MEGA 7.0.18 (Kumar et al., 2016) using default parameters for ClustalW (Larkin et al., 2007) alignment. Phylogenetic trees for the whole-genome were created using BEAST v1.10.4 (Suchard et al., 2018). Classification was performed by considering the support of nodes by posterior probability values in relation to specific additionally identified substitutions and the reference genomes. Based on the whole-genome tree, eleven phylogenetic groups were identified (Van Poelvoorde et al., 2021): “Group 3C2a” (*n* = 4), “Group 3C2a1” (*n* = 26), “Group 3C2a1(2)” (*n* = 62), “Group 3C2a1a” (*n* = 25), “Group 3C2a1a (2)” (*n* = 37), “Group 3C2a1b” (*n* = 20), “Group 3C2a2” (*n* = 9), “Group 3C2a3” (*n* = 59), “Group X” (*n* = 8), “WGX” (*n* = 1) and “WGY” (*n* = 2) (Figure 1). Based on this whole-genome phylogenetic tree, the viral genetic background was taken into account by grouping samples in phylogenetic groups since less diversity exists within these groups. The phylogenetic classification groups together samples with the same characteristic mutations for that particular phylogenetic group. These characteristic mutations make up the viral genetic background. To retain statistical power for the number of available samples (because too few samples were available for some phylogenetic groups to perform sound statistical inference), individual phylogenetic groups were combined based on an objective criterion by considering their sequence identity. The terms “clade” and “group” refer specifically to grouped samples using either the WHO/ECDC recommendations or our classification method, respectively. The exact sequence identity threshold was calculated from the WHO/ECDC clades, which include “Clade 3C2a1” (*n* = 170), “Clade 3C2a2” (*n* = 9) and “Clade 3C2a3” (*n* = 59). However, as only nine Belgian samples belonged to “Clade 3C2a 2”, these were grouped with “Clade 3C2a1” because the sequence identity showed that these ten samples are most similar to samples from “Clade 3C2a1” with a minimal sequence identity of 98.81% versus a sequence identity of 98.67% compared to “Clade 3C2a3”. The sequence identity between the concatenated genome sequences of all samples was calculated using the “Ident and Sim” tool (Stothard, 2000). A percent identity cut-off of 98.81% was selected for combining phylogenetic groups. This resulted in classifying the 253 samples into three groups (Figure 1). “Phylogenetic Group X” consisted of 190 samples from the following individual phylogenetic groups: “Group 3C2a1”, “Group 3C2a1(2)”, “Group 3C2a1a”, “Group 3C2a1a (2)”, “Group 3C2a1b”, “Group 3C2a2”, “Group X”, “WGX”, and “WGY”. The second group of 59 samples all belonged to the phylogenetic group “Group 3C2a3”. The third group of 4 samples all belonged to the phylogenetic group “Group 3C2a,” but were not retained for further analysis due to the limited number of samples. A list of the amino acid substitutions that were found in each sample of each respective group is provided in Supplementary Table 2: AA_MUT_Phylo.

In order to compare results from the Belgian strains with the international context, a local Nextstrain instance (Hadfield et al., 2018), allowing light-weight phylogenomics, was built using the in-house sequenced samples complemented with GISAID sequences. Only samples that included the whole genome, patient sex, age information, and that were directly sequenced (i.e., no passaging in cells or eggs) were used, resulting in 14,157 samples. All sequences were aligned with CLC Genomics Workbench 20.0.2 with default parameters and untranslated regions were stripped on both sides retaining only the protein-coding parts. Aligned segments were concatenated into a single sequence for all samples. Only sequences with less than three gaps and/or ‘N’ characters were retained (Supplementary Table 1). To create the local instance, the same steps were taken as described previously (Van Poelvoorde et al., 2021). The Belgian samples were previously designated to their phylogenetic groups (Van Poelvoorde et al., 2021). Finally, GISAID samples that clustered with these Belgian samples were assigned to the same phylogenetic group.

### Inference of Associations With Patient Data

Statistical data analyses were performed using R-software (RStudio Version 1.0.153; R Version 3.6.1). For the “general approach”, the two-sided Fisher’s exact test was used to assess the association between the variables obtained from the clinical patient files and amino acid mutations from all samples that were identified in comparison to A/HongKong/4801/2014. These variables that were used in the two-sided Fisher’s exact test were obtained from the clinical patient files and include patient age (categorized into < 15, 15-59, and ≥ 60), sampling date, sex, vaccination status, the use of antibiotics, presence of comorbidities, disease severity (classified into mild, moderate, and severe). Disease severity is based on the one hand on the surveillance system: ILI (mild) and SARI (moderate + severe); and on the other hand on the absence (moderate) or presence (severe) of severity indicators. The distinction between moderate and severe among SARI patients is made based on the severity indicators. Disease severity indicators (death, stay in the ICU and advanced respiratory support) were also considered separately. Multiple testing correction was applied by employing the Benjamini-Hochberg method [53] and controlling the False Discovery Rate (FDR) at 5%. The variance inflation factor (VIF) was used to measure the amount of collinearity between mutations when inserted as a set of multiple regression variables.

Because of the overall high genetic diversity within the sequenced samples (Van Poelvoorde et al., 2021), the importance of the viral genetic background was explored. For this “approach considering the viral genetic background”, the same statistical analysis was conducted to detect mutations linked to the patient data within the two groups, i.e., the previously described “Phylogenetic Group X” and “Group 3C2a3.” For statistically significant associations identified during the univariate analysis, generalized linear regression with a binomial family distribution was used to identify confounding mutations and evaluate the effect modification. Confounding factors were identified by adding potential risk factors (other patient data) to the model. The effect modification was evaluated by adding interaction terms to the model. Additionally, the effect size was defined as an odds ratio as estimated by using a logistic regression analysis for the association in question.

**FIGURE 2:**
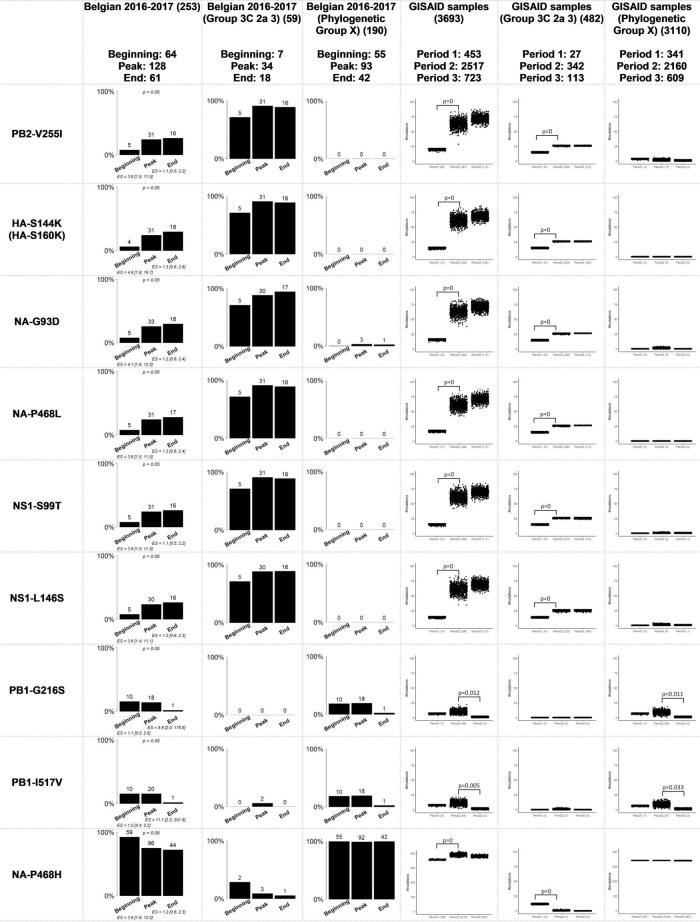
Comparison of the Belgian influenza samples with samples from the GISAID database for mutations that were considered significantly related to the sampling period. The distribution of samples in the groups “Group 3C 2a 3” and “Phylogenetic Group X” is provided for the significant results after running the Fisher’s exact test with FDR correction when the viral genetic background is not taken into account. “Phylogenetic Group X” includes “Group 3C2a1”, “Group 3C2a1(2)”, “Group 3C2a1a”, “Group 3C2a1a (2)”, “Group 3C2a1b”, “Group 3C2a2”, “Group X”, “WGX” and “WGY”. In the graphs representing the situation in Belgium, above the bars the number of samples that had this mutation are indicated. In the graphs representing the samples from GISAID, the number of samples that possessed this mutation are indicated below the chart. The magnitude of the significant association is defined by the effect size (ES) and its confidence interval. The resulting *p*-value of the Fisher’s exact test with FDR correction for the samples from the Belgian dataset is indicated above bar charts for which significant associations were found. The *p*-values of the permutation tests performed for the GISAID samples are indicated above the boxplots.

The GISAID database contains information on patient sex and age, and sampling date. Strains from the WHO-defined clade “3C3a” were excluded from this analysis based on the custom-built Nextstrain instance of 10,583 samples because these strains were genetically too distant from the Belgian sequenced samples (Data Sheet 2: Supplementary Figure 1 and Supplementary Table 2: GISAID_Samples). These samples were used for the general approach as well as for the approach considering the viral background, which resulted in 8,796 samples belonging to “Phylogenetic Group X” and 831 samples belonging to “Group 3C2a3”. Samples collected between April 2016 and September 2017 were attributed to three “period groups” and these period groups each included samples from both the Northern and Southern hemispheres. The first period comprised the end of the 2015-2016 Northern hemisphere influenza season in April 2016 until week 45 of 2016 (453 samples). The second period comprised samples until week 16 of 2017, when less than 10% of lab tests were positive for influenza in Europe according to ECDC (European Centre for Disease Prevention and Control [ECDC], 2020a) (2,517 samples). The third period comprised samples until the end of September 2017 (723 samples). The first and third periods were predominated by samples from the Southern hemisphere, while the second period was predominated by samples from the Northern hemisphere, corresponding with their respective flu seasons. Because the number of genome sequences per period group varied, permutation analyses were performed to correct for sample size. In total, 1,000 subsets of 430 genome sequences were randomly selected for every period group by sampling with replacement (Figure 2 and Data Sheet 2: Supplementary Figure 3) using a sample size of 95% of the smallest group. It was then assessed if the same significantly associated trends could be distinguished compared to the trends observed within the Belgian samples by performing a two-sided permutation test at a significance level of 0.05.

Finally, it was evaluated whether the mortality was significantly higher within the group of patients suffering from renal insufficiency using a Chi-square Goodness of Fit Test. All analysis scripts and results are provided in Data Sheet 1. Input files and detailed results are provided in Supplementary Table 2.

## Results

### Significant Associations Between Viral Mutations and Patient Data

The aim was to identify potential associations between specific amino acid mutations in the influenza genome and available patient data in a cohort of 253 influenza patients (Table 1). Table 2 lists an overview of the identified statistically significant associations and previously described effects of these mutations reported in literature. Significant associations were detected between specific mutations and the sampling period, and the patient sex (Supplementary Table 2: AAMut Fisher + FDR). Nine mutations were linked to their sampling period and their presence in the circulating strains significantly varied (two-sided Fisher’s exact test with FDR correction of 5%) during the season with an effect size ranging from 1.1 to 11.1 (Figure 2). The mutations PB2-V255I (adjusted *P* = 0.05), HA-S144K (adjusted *P* = 0.05), NA-G93D (adjusted *P* = 0.05), NA-P468L (adjusted *P* = 0.05), NS1-S99T (adjusted P = 0.05), and NS1-L146S (adjusted P = 0.05) emerged over time, whereas the mutations PB1-G216S (adjusted *P* = 0.05), PB1-I517V (adjusted *P* = 0.05), and NA-P468H (adjusted *P* = 0.05) decreased. At position 468 in the NA segment, the mutations NA-P468L and NA-P468H emerged and decreased, respectively, throughout the season. The VIF-analysis demonstrated that viruses containing the PB1-G216S mutation, often co-occurred with the PB1-I517V mutation. These mutations were most often observed in “Group 3C2a1a(2)” (Data Sheet 2: Supplementary Figure 4). Additionally, samples containing the PB2-V255I mutation often possessed the other emerging mutations (HA-S144K, NA-G93D, NA-P468L, NS1-S99T, and NS1-L146S). These mutations were most often observed in “Group 3C2a3” (Data Sheet 2: Supplementary Figure 3). For these associations, particular confounding factors existed, i.e., other variables influencing the correlation between the mutation and the patient data, that could not be excluded. These factors included vaccination status, antibiotics use, surveillance system, and/or stay in the ICU (Supplementary Table 2: Effect Size). Ten mutations were observed to be significantly more present in either male or female patients (Data Sheet 2: Supplementary Figure 2) (detailed results in Supplementary Table 2: AAMut Fisher + FDR).

**TABLE 2:** Statically significant associations found between patient data and amino acid mutations in the whole genome.

AA substitution	Functional site	Amino acid properties	Previous descriptions	Citations
**General approach**				
**Sampling period**				
PB2-V255I	NP binding site Wang et al., 2016	Size Medium → Large	Association between this mutation and patients that were not vaccinated.	Simon et al., 2019
			No association with a significant change in pathogenicity in A(H1N1) and A(H3N2).	Andrés et al., 2015; Cai et al., 2020
			Increase in pathogenicity due to this mutation in combination with seven other residues (H15R, N23S, T27I, K53R, L58S, R75H, H75L) in A(H1N1)	Koçer et al., 2015.
HA-S144K	Receptor-Binding domain Tzarum et al., 2015 Epitope region A Jorquera et al., 2019	Charge Neutral → Basic Size Very small → Large	Association between this mutation and patients that were not vaccinated.	Simon et al., 2019
			Link with low vaccine effectiveness.	Trebbien et al., 2017
NA-G93D	Head: Enzyme active site and calcium binding domain, which stabilizes the enzyme structure at low pH values Hirst, 1942; Gottschalk, 1957; Webster and Laver, 1967; Palese and Compans, 1976; Varghese et al., 1983; Russell et al., 2006	Polarity Non-polar → Polar Charge Neutral → Acidic Size Very small → Small	Association between this mutation and patients that were not vaccinated.	Simon et al., 2019
NA-P468L	Head: Enzyme active site and calcium binding domain, which stabilizes the enzyme structure at low pH values Hirst, 1942; Gottschalk, 1957; Webster and Laver, 1967; Palese and Compans, 1976; Varghese et al., 1983; Russell et al., 2006	Size Small → Large	No studies were found.	
NS1-S99T	Effector domain Hale et al., 2008	Size Very small → Small	Association between this mutation and patients that were not vaccinated.	Simon et al., 2019
NS1-L146S	Nuclear export signal Hale et al., 2008	Polarity Non-polar → Polar Hydropathy Hydrophobic → Hydrophilic Size Large → Very small	Association between this mutation and patients that were not vaccinated.	Simon et al., 2019
PB1-G216S	Nuclear Localization Signal Ping et al., 2011	Polarity Non-polar → Polar	A(H1N1) viruses with PB1-216G have an increased adaptability and enhancement of viral epidemiological fitness, probably due to a low-fidelity replicase. PB1-216S viruses showed a higher pathogenicity in mice in comparison to PB1-216G viruses and PB1-216S viruses had a lower mutation potential.	
PB1-I517V	Not described	Size Large → Medium	This position in the H3N8 virus was identified as undergoing changes due to selective pressure during host shifts from birds to humans. This mutation in a A(H1N1)pdm09 viral background was discovered in a highly complementary region between PB1 and HA and leads to an enhancement of the complementarity and consequently better binding. In the mammalian host due to a more restricted conformation, this apparent neutral mutation is located near conserved motifs that are responsible for protein folding and this effect suggests that the mutation leads to a better compatibility with H1 in the human host.	Tamuri et al., 2009; Nilsson, 2017; Lin et al., 2019
NA-P468H	Head: Enzyme active site and calcium binding domain, which stabilizes the enzyme structure at low pH values Hirst, 1942; Gottschalk, 1957; Webster and Laver, 1967; Palese and Compans, 1976; Varghese et al., 1983; Russell et al., 2006	Polarity Non-polar → Polar Charge Neutral → Positive Hydropathy Hydrophobic → Hydrophilic Size Small → Medium	Association between this mutation and patients that were vaccinated.	Simon et al., 2019
			It was demonstrated that P468H has become fixed in A (H3N2) viruses circulating since 2016. This mutation contributed to NA antigenic drift in relation to the vaccine strain Hong Kong/4801/2014. There is further research needed to understand the role of the mutation, because residue 468 is not essential for binding antibodies.	Wan et al., 2019

**Approach considering THE VIRAL BACKGROUND**				
**Renal Insufficiency**				

PB2-R299K	Not described	Not applicable	It was demonstrated in A(H1N1)pdm09-infected mice that K299 is conserved, which raises the possibility that it plays some role in the adaptation to the mammalian host and might also link to the heterogeneity in A (H1N1)pdm09.	Farooqui et al., 2012
			It has been observed that eleven amino acid mutations, including PB2-R299K, in A (H3N2) occurred between the influenza virus strains in the 2016-2017 winter season and 2017 summer season. These mutations were correlated to temperature sensitivity and viral replication, because the 2016-2017 winter season viruses were significantly restricted at 39°C. Although this mutation was identified, it had little influence on the polymerase activity at different temperatures.	Wei et al., 2018
PB2-K340R	Cap binding Guilligay et al., 2008; Fodor, 2013	Conservative	PB2-K340R was introduced in a PR8-derived recombinant virus A (H1N1) and there was no significant increase in polymerase activity.	Lee et al., 2017
			It has been observed that eleven amino acid mutations, including PB2-K340R, in A (H3N2) occurred between the influenza virus strains in the 2016-2017 winter season and 2017 summer season. These mutations were correlated to temperature sensitivity and viral replication, because the 2016-2017 winter season viruses were significantly restricted at 39°C. Although this mutation was identified, it had little influence on the polymerase activity at different temperatures.	Wei et al., 2018
HA-K92R	Epitope Region E (Eshaghi et al., 2014)	Conservative	This mutation was confirmed in this study as specific for the HA cluster 3C2a1b.	European Centre for Disease Prevention and Control [ECDC], 2020b
HA-H311Q	Epitope region C Kawakami et al., 2019	Charge Positive → Neutral	This mutation was confirmed in this study as specific for the HA cluster 3C2a1b.	European Centre for Disease Prevention and Control [ECDC], 2020b
NP-V197I	Cytotoxic T lymphocyte (CTL) epitopes Galiano et al., 2012	Size Medium → Large	This mutation in a A(H3N2) virus is located in known virus CTL epitopes and they may confer a higher efficiency of escape from CTL-mediated immune responses.	Galiano et al., 2012

*Functional sites and properties of amino acid changes are also presented. Volume categories for size are divided in “very small” [60-90 A^3^], “small” [108-117 A^3^], “medium” [138-154 A^3^], “large” [162-174 A^3^] and “very large” [189-228 A^3^]. Finally, the description of the mutation was included if available in the literature. All five mutations related to renal insufficiency when considering the viral genetic background were found within “Phylogenetic Group X”. “Phylogenetic Group X” includes “Group 3C2a1”, “Group 3C2a1(2)”, “Group 3C2a1a”, “Group 3C2a1a (2)”, “Group 3C2a1b”, “Group 3C2a2”, “Group X”, “WGX” and “WGY”.*

### Significant Associations Between Mutations and Patient Data When Samples Are Stratified According to the Phylogenetic Clade

It was previously shown that similar mutations can affect viral genes in different and sometimes even contradictory ways (Visher et al., 2016; Lyons and Lauring, 2018). These variations can possibly be attributed to potential effects related to the highly diverse genetic background of the A (H3N2) subtype (Chandler et al., 2013). The viral background was taken into account while using phylogenetic classification based on a whole-genome tree (Van Poelvoorde et al., 2021). The 253 samples were classified into three groups (Figure 1). “Phylogenetic Group X” (*n* = 190), “Group 3C2a3” (*n* = 59) and “Group 3C2a” (*n* = 4), but the latter was not retained for further analysis due to the limited number of samples. We compared the previously found associations using the general approach in this study (Results 3.1) within “Phylogenetic Group X” and “Group 3C2a3” separately, by taking the viral genetic background in account based on the phylogenetic groups.

**FIGURE 3:**
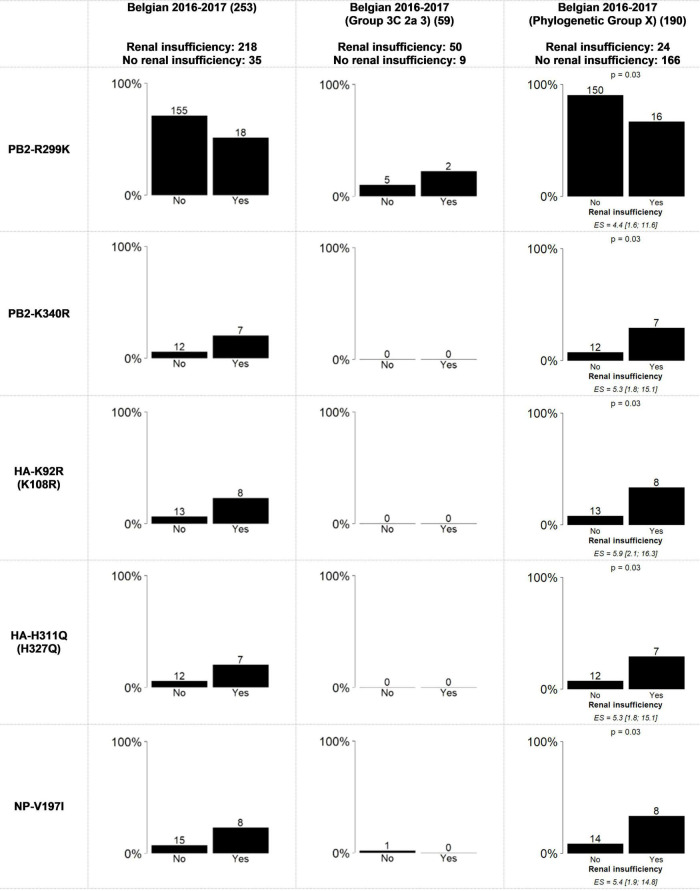
Statically significant results using the Fisher’s exact test with FDR correction for the association between renal insufficiency and amino acid mutations in the whole genome from all of the samples and “Phylogenetic Group X” and “Group 3C2a3”. “Phylogenetic Group X” includes “Group 3C2a1”, “Group 3C2a1(2)”, “Group 3C2a1a”, “Group 3C2a1a (2)”, “Group 3C2a1b”, “Group 3C2a2”, “Group X”, “WGX” and “WGY”. The bar graphs represent the percentage of samples per variable of the patient data that have the mutation. On top of the bars the number of samples that had this mutation are indicated. The magnitude of the significant association is defined by the effect size (ES) and its confidence interval. The resulting p-value of the Fisher’s exact test with FDR correction is indicated above bar charts for which significant associations were found.

Associations between the previously identified mutations and the sampling period (Figure 2), that were significant using the general approach (Results 3.1), presented similar trends but were no longer statistically significant. For the mutations found to be significantly more present in male or female patients, the same trends were not observed within the groups when the viral genetic background was considered (Data Sheet 2: Supplementary Figure 2). Importantly, unequal distribution between male and female patients was observed (Van Poelvoorde et al., 2021) as each of these mutations was observed almost exclusively in either “Phylogenetic Group X” (Female = 102; Male = 81) or “Group 3C2a3” (Female = 18; Male = 40). Additionally, five mutations within “Phylogenetic Group X” were significantly associated with renal insufficiency (Supplementary Document 3: AAMut Fisher + FDR (PHYLOX)) (two-sided Fisher’s exact test with FDR correction of 5%). Table 2 presents an overview of these mutations and their previously described effects in the literature. PB2-R299K (adjusted *P* = 0.03), was significantly more present in samples from patients without renal insufficiency. PB2-K340R (adjusted *P* = 0.03), HA-K92R (adjusted *P* = 0.03), HA-H311Q (adjusted *P* = 0.03), and NP-V197I (adjusted P = 0.03), were significantly more detected in patients suffering from renal insufficiency (Figure 3). The VIF-analysis demonstrated samples containing the PB2-K340R mutation, often co-occurred with the HA-K92R, HA-H311Q, and NP-V197I mutations. Most of these mutations are observed within “Group 3C2a1b” (Data Sheet 2: Supplementary Figure 6 and Supplementary Table 2: Mutations per group). To demonstrate the limited effect of reassortment on the associations with renal insufficiency, the same analysis, namely a two-sided Fisher’s exact test with FDR correction (5%), was performed for each segment tree. In most cases, the associations related to the renal insufficiency remained significant, suggesting that these associations were not related to reassortment (Supplementary Table 2: Segment Renal).

Although the exact stage of chronic renal insufficiency was not specified in our dataset and most patients suffered from other chronic diseases and/or were elderly, seven out of 35 patients with this condition did not survive, which is significantly more than in the total dataset (Chi-square Goodness of Fit Test, *p* = 0.005). For more detailed results, see the Supplementary Table 2.

### Evaluation of Significantly Associated Mutations From the Belgian Samples in an International Context

To evaluate significant associations observed in the Belgian dataset in a more global context, the Belgian samples were supplemented with samples from the same subtype for which patient information was available in the GISAID database (patient age, sex, and sampling date). The significant associations observed in the Belgian study were compared to the results in the GISAID database for both the general approach and the one considering the viral genetic background. Although some bias may be introduced by (i) a different selection criterion to choose the isolates to sequence by the different laboratories; (ii) different sampling population (patient) sizes, our observations are the following:

Regarding the sampling date (Figure 2), all of the mutations related to the sampling period, except for NA-P468H, showed the same significantly associated trend over time as observed in the Belgian study. Additionally, the observed trends when considering the viral genetic background were significant in contrast to the Belgian samples, probably due to the increase in sample size. It is therefore possible to partially extrapolate the results of the Belgian study to a global level.

Regarding patient sex, the samples from the GISAID database did not follow the same trends as the Belgian influenza samples for both the general approach and the one considering the viral genetic background (Data Sheet 2: Supplementary Figure 2). It should be noted that in contrast to the Belgian influenza samples, the number of male and female patients extracted from the GISAID database, was more equally distributed across the groups, namely “Phylogenetic Group X” (Male = 4156; Female = 4639) or “Group 3C2a3” (Male = 431; Female = 397).

## Discussion

Influenza surveillance is the basis for determining the seasonal influenza vaccine composition. Current conventional influenza vaccines are still largely based on technology from the 1940s relying on the replication of influenza in embryonated eggs and focuses on the HA segment (Milián and Kamen, 2015). However, next-generation vaccines also focus on other parts of the genome, consequently to track mutations across the whole genome becomes important for such vaccine candidates. Moreover, WGS enables the detection of mutations across all eight segments of the influenza genome, allowing the evaluation of associations between the patient data and mutations located on the whole viral genome instead of solely the HA segment. It is important for the influenza surveillance to provide information to national influenza prevention and control programs about the severity, impact, and timing of seasonal epidemics.

In this study, mutations were identified using WGS data of influenza A (H3N2) samples collected in the context of the influenza surveillance in Belgium. They were used in order to explore potential associations between mutations positioned across the whole genome and patient characteristics as well as other metadata. Due to the limited number of samples, that is often the case for national surveillance, it was verified, when it was possible, whether the observations at the Belgian level correspond to trends at an international level using the GISAID database. For example, significant increase or decrease over the sampling periods was observed for nine mutations located across the A(H3N2) genome during the Belgian 2016-2017 influenza season. Comparison with the GISAID database showed the same significantly associated trends worldwide for these mutations, except for NA-P468H. These mutations can probably be attributed to the fast evolutionary dynamics of influenza A (H3N2) (Allen and Ross, 2018). Throughout the outbreak season, it is relevant to follow trends of emerging and disappearing mutations over the whole genome with respect to the vaccine strain, as these mutations may lead to antigenic drift from the vaccine strain. Currently, only the HA and NA segments are updated in the vaccine, the HA and NA mutations and their evolution over time should therefore be considered for the vaccine composition for the next influenza season. The vaccine strain of the 2017-2018 influenza season, which is also A/Hong Kong/4801/2014 (European Medicines Agency, 2018), and subsequent years did not take these into account, which can be a partial explanation for the observed low vaccine efficacy in that season. The importance of following the emergence or decrease of mutations with respect to selecting the appropriate vaccine strain can be illustrated by the HA-S144K mutation, which significantly increased during the Belgian 2016-2017 influenza season. HA-S144K together with HA-N121K and HA-T135K were previously associated with outbreaks in the Northern hemisphere and suboptimal vaccine effectiveness (Glatman-Freedman et al., 2017; Melidou et al., 2017; Nastouli et al., 2017; Skowronski et al., 2017; Trebbien et al., 2017; Tsou et al., 2017). Noteworthy, although not significant (potentially due to the limited number of samples), HA-N121K and HA-T135K also increased during the influenza season in the Belgian surveillance (results not shown).

The substantial diversity observed within the patient derived A(H3N2) isolates during the Belgian 2016-2017 season (Van Poelvoorde et al., 2021) offered the opportunity to explore whether considering the viral genetic background by stratifying the samples according the phylogeny has an effect on the detection of new associations. Importantly, when stratifying the sample according the phylogeny, we discovered associations between the occurrence of certain mutations in the A (H3N2) viruses and patient data. In our Belgian study, in particular associations related to renal insufficiency were detected after genetic stratification. In addition to severity indicators, it could be relevant for patient management to explore associations with comorbidities, including renal insufficiency. Influenza contributes to higher mortality in patients suffering from End-Stage Renal Disease, which is the last stage of chronic renal insufficiency or chronic kidney disease (Gilbertson et al., 2019). Patients with chronic renal insufficiency also often suffer from other diagnosed or undiagnosed risk factors possibly resulting in a poor outcome when infected with the influenza virus (Kausz and Pahari, 2004; Choudhury and Luna-Salazar, 2008; Demirjian et al., 2011). Four and one mutation(s) were detected to be significantly more likely to be present and absent in patients suffering from renal insufficiency, respectively, both within “Phylogenetic Group X”. It could be speculated that these mutations associated with renal insufficiency could be the result of a weakened immune system. To support this finding, it should be emphasized that the nasopharyngeal swabs that were obtained from the 35 patients with renal insufficiency were taken in hospitals across Belgium, and were not restricted to one of the sampling periods that were defined in this study (beginning of the influenza season, peak of the influenza season and end of the influenza season), and no evidence of epidemiological linkage could be found (Supplementary Table 2: Metadata).

However, we cannot exclude other confounding factors that lead to these associations. Except for the vaccination status, if available, the immune status of the patient was not included in the analysis as this information was not available. These associations related to renal insufficiency could unfortunately not be confirmed with a larger number of samples from the GISAID database because this database does not contain this type of patient information. Regarding the results of this proof of concept study, it is important that the occurrence of these mutations be examined in the following years from the surveillance system in Belgium and other countries to learn if these associations can be confirmed.

A collection containing a small number of samples like in this study as limitations. Stratification according to the phylogeny has the inconvenience to further reduce this number. Indeed, on the one hand the significance of some associations obtained within the larger group may disappear due to a lack of power for the statistical analysis. On the other hand, the small number of samples and the multivariable analysis may introduce bias leading to a “false” association. Therefore, it is advised to have a confirmation with a larger dataset if possible. In this study, this was illustrated by the fact that the significant associations related to the sampling period, observed without stratification, was following the same trends but did not result in significant associations anymore when considering the viral background. This is probably due to the limited number of samples, which was confirmed using a larger dataset of GISAID while considering the viral genetic background. Using the GISAID dataset, the associations regarding the sampling period became significant for the same groups and trends.

Another limitation was related to the ten mutations identified to be significantly related to the sex of the patient with the general approach. However, this trend was not confirmed when using the GISAID database. In fact, conflicting results (opposite trends) were observed in comparison to the general approach for the mutations related to sex when the viral genetic background was considered. These results considering the viral genetic background could also not be confirmed using the sequences from the GISAID database for both the general approach and the approach taking into consideration the viral genetic background. A probable cause of this inconsistency between the general approach, the approach considering the viral genetic background and the GISAID database could be the unequal distribution of male and female patients in the Belgian dataset over the different phylogenetic groups causing a gender sampling bias. In Data Sheet 2: Supplementary Figure 2, the unequal distribution is explained more in detail. In this study, the number of samples was limited to 253 samples due to the current infrastructure and cost of sequencing.

This proof of concept study highlights the power that WGS sequencing of influenza may offer especially when using a stratification taking into account the viral genetic background. It shows also the limitation linked of analyzing a small number of samples. However, such size is a reality for several countries as it is already challenging to acquire the necessary funds to simply switch from Sanger sequencing the HA and NA segments to WGS in routine surveillance. Therefore, it would be of great benefit to perform such type of analysis at a European or international level using more samples to reduce the effect of sampling bias and to have more statistical power to find other associations.

The analysis of the GISAID database in this study has demonstrated that using a larger dataset could help to confirm the trends observed with a relatively limited number of samples within countries like Belgium. Also, when using genomic data, large sample sizes are needed because many mutations were included in the analysis, leading to a reduction of the statistical power due to multiple testing correction. This may be particularly crucial when using the approach taking into account the viral genetic background and working with smaller groups. In this context, a large database available to the scientific community containing genomic data with a larger set of patient data is important to be constructed. This is currently only in place to a limited extent. The WHO maintains a list of mutations linked to resistance of neuraminidase inhibitors (WHO, 2016). FluSurver is an application utilizing an in-house database of curated literature annotations for mutational effects associated with antibody escape, antigenic drift, host receptor specificity, and drug resistance. Broadening the scope of such resources could allow exploring associations between particular mutations and (other) phenotypic effects and patient data (Maurer-Stroh et al., 2020). GISAID maintains sequence data worldwide and could be useful to investigate if the effects of particular mutations have also been observed in other genome sequences sampled at other geographical locations and during other influenza seasons. However, available patient information in GISAID is currently mostly limited to the age and sex of the patient and the sampling date (Shu and McCauley, 2017; Velazquez et al., 2020). In addition, the GISAID licenses should be less restrictive, allowing their data to be used more easily. The construction and the use of a database with a large dataset coming from samples selected and sequenced by different laboratories and different countries is also challenging. Indeed, this implies the need for having a common, standardized approach to collect and manage data within different laboratories or at least to provide a detailed description of the methodology used to collect the sample and the patient data in order to avoid potential bias which could result in erroneous conclusions. For example, although SARS-CoV-2 was the most sequenced virus, due to the lack of harmonization between countries it remains difficult to draw conclusions whether certain SARS-CoV-2 mutations are related to disease severity, vaccination or other patient data mutations related to the season. Calling for a new approach to data management could enable faster solutions and improve the worldwide response of the scientific community resulting in a better surveillance.

In conclusion, the results of this study, identifying associations between the patient data and viral mutations that were not only present in the HA segment, highlight the importance of tracking mutations across the entire influenza genome. Furthermore, this study is used as a proof of concept to demonstrate how to work with real-world data coming from National Reference Centers when WGS is implemented in routine surveillance. In addition to disease severity and vaccination status, other patient data was included in this study such as age, severity indicators (stay in the ICU, death, need for invasive respiratory support, ARDS and ECMO), comorbidities (renal insufficiency, cardiac, neuromuscular and respiratory diseases, hepatic insufficiency, diabetes, and, immunodeficiency) and sampling date. This study detected associations between particular mutations and the sampling period that can be important to take into account for vaccine strain selection and clinical management of infected patients. Moreover, this study investigated the possible effect of the viral genetic background on the association between mutations and patient data and proposed a new approach based on stratification using phylogenetic groups. Using this approach, five additional mutations significantly associated with renal insufficiency were detected, indicating the potential or even necessity to take the viral genetic background of the virus into account by considering its phylogeny. Therefore, the viral genetic background could play an important role in inferring associations between genomic and patient data.

## Data Availability Statement

The datasets presented in this study can be found in online repositories. The names of the repository/repositories and accession number(s) can be found below: https://www.gisaid.org/, EPI_ISL_415199 to EPI_ISL_415452; https://www.ncbi.nlm.nih.gov/, PRJNA615341.

## Ethics Statement

The studies involving human participants were reviewed and approved by a central ethical committee and the local ethical committees of each participating hospital approved the SARI surveillance protocol (reference AK/12-02-11/4111; in 2011: Centre Hospitalier Universitaire St-Pierre, Brussels, Belgium; from 2014 onward: Universitair Ziekenhuis Brussel, Brussels, Belgium). Written informed consent to participate in this study was provided by the participants’ legal guardian/next of kin. Written informed consent was obtained from the minor(s)’ legal guardian/next of kin for the publication of any potentially identifiable images or data included in this article.

## Author Contributions

NR, KV, IT, XS, SD, and SV: conceptualization. NR: project administration and funding acquisition. IT, LV, and NV: data curation. IT, SV, SD, and KV: resources. LV and KV: formal analysis. LV: investigation and visualization. LV and NR: writing – original draft preparation. NR, KV, and IT: supervision. All authors wrote, reviewed, and edited the manuscript.

## Conflict of Interest

The authors declare that the research was conducted in the absence of any commercial or financial relationships that could be construed as a potential conflict of interest.

## Publisher’s Note

All claims expressed in this article are solely those of the authors and do not necessarily represent those of their affiliated organizations, or those of the publisher, the editors and the reviewers. Any product that may be evaluated in this article, or claim that may be made by its manufacturer, is not guaranteed or endorsed by the publisher.
