# Supplementary material for: Whole-Genome Sequence Approach and Phylogenomic Stratification Improve the Association Analysis of Mutations With Patient Data in Influenza Surveillance
Source: Front Microbiol. 2022 Apr 19;13:809887. doi: 10.3389/fmicb.2022.809887 (PMC9063638; doi:10.3389/fmicb.2022.809887)
Supplement: Supplementary file 1 [file Data_Sheet_1.PDF]

Contents

Are mutations linked to specific host characteristics 3
Read in data 4
Metadata 4
List of mutations 5
Univariate analysis: GWAS 5
Amino acids 5
Link between mutations 8
Sex 8
Sampling Period 9
Logistic regression 12
Host characteristics 12
Confounding factors and effect modification 13
Effect size 15
Are mutations within phylogenetic groups linked to specific host characteristics 18
Read in data 19
Metadata 19
List of mutations 20
Univariate analysis: GWAS 21
Link between mutations 23
Logistic regression 24
Host characteristics 24
Confounding factors and effect modification 25
Effect size 27
Comparison to GISAID samples 29
Global context: Read in data 29
Metadata 29
Sequences 30
Match metadata and sequences 31
Mutations 32
Sampling Period 32
Mutations: Sex 33
Boxplot 35

Sampling Period 35
Sex 40
Proportion fatalities in renal insufficiency patients 43

Are mutations linked to specific host characteristics

Input files: All the input files and results are collected in one xls file in Supplementary Document S3

Input files:

- Set of nucleotide mutations with mutations selected based on coverage (>100) and frequency (MAF>5%)
- Set of amino acid mutations with prioritized mutations based on coverage (>100), frequency (MAF>5%) and change in amino acid
- Metadata file

Frequency of mutations >5% and <95%

Output:

- Univariate GWAS (including correction for multiple testing)
- Binary outcome: Fisher's exact test + FDR correction
- Multinomial outcome (2x3 tables): Fisher's exact test + FDR correction

---

```
library(adegenet)
library(glmnet)
library(ShortRead)
library("DECIPHER")
library(msa)
library("FactoMineR")
library("factoextra")
library(rcompanion)
library(RVAideMemoire)
library(fmsb)
library(reshape2)
library(tidyr)
library(dplyr)
library(ggplot2)
library(tibble)
library(flextable)
library(car)
library("readxl")
```

Read in data

Metadata

- 160 **SARI** samples
- 93 **ILI** samples

```
metadata= read_excel("2. Supplementary File - Input files and results 
statistical test.xlsx", sheet = "METADATA ", header = TRUE, sep = ",", quote 
= "\"", dec = ".")
# n=253

#ILI=0; SARI=1
#Male=0; Female=1
#No=0; Yes=1
#Alive=0; Deceased=1
#Beginning=0; Middle=1; End=2
#<15=0; 15-59=1; >=60=2
#Mild=0; Moderate=1; Severe=2


metadata$Reference <- (as.character(metadata$Reference))

metadata[metadata == "16-IG0682"] <- "16-IG-0682"
metadata[metadata == "16-IG0711"] <- "16-IG-0711"
metadata[metadata == "16-IG0712"] <- "16-IG-0712"
metadata[metadata == "16-IG0720"] <- "16-IG-0720"

metadata[] <- lapply(metadata,factor)
```

List of mutations

Subset 1: prioritized mutations: *amino acids*

- Non-synonymous amino acids
- Filtering frequency 5-95%
- Coverage >100
- Biallelic and triallelic –> triallelic: added extra variable

Subset: amino acids

```
aamut= read_excel("2. Supplementary File - Input files and results
statistical test.xlsx", sheet = "Non-Synonymous aa", header = F, sep = ",",
quote = "\"", stringsAsFactors=F)
aamut=t(aamut)
colnames(aamut)=aamut[1,]
rownames(aamut)=aamut[,1]
aamut=aamut[-1,-1]
aamut=as.data.frame(aamut)

# all aa mutations coverage of >100
aamut[] <- lapply(aamut,factor)
metadata<- metadata[match(rownames(aamut),
as.character(metadata$Reference)),]
metadataaamut <- cbind(metadata,aamut)
```

Univariate analysis: GWAS

Amino acids

```
metadata<- metadata[match(rownames(aamut),
as.character(metadata$Reference)),]

varlist2 <- c("metadata$Surveillance", "metadata$Sex", "metadata$ca_vacci",
"metadata$ca_status", "metadata$ca_antibio", "metadata$asthma_respi",
"metadata$cardio", "metadata$obesity", "metadata$diabete", "metadata$renal",
"metadata$immuno", "metadata$neuro", "metadata$hepato", "metadata$icuever")

varlist3 <- c("metadata$Period", "metadata$Age.cat",
"metadata$MildModerateSevere")

varlist <- c("Surveillance","Sex", "ca_vacci", "ca_status", "ca_antibio",
"asthma_respi", "cardio", "obesity", "diabete", "renal", "immuno", "neuro",
"hepato", "icuever","Period", "Age.cat", "MildModerateSevere")


aamut.fun2 <- (paste("apply(aamut,2,function(e)
fisher.test(table(factor(e,levels=c(0, 1)), factor(", varlist2,", levels=c(0,
1))))$p.value)", sep=""))
aamut.fun3 <- (paste("apply(aamut,2,function(e)
```

```
fisher.test(table(factor(e,levels=c(0, 1)), factor(", varlist3,", levels=c(0,
1, 2))))$p.value)", sep=""))
aamut.fun <- c(aamut.fun2,aamut.fun3)
aamut.fisher <- lapply(aamut.fun, function(x) {eval(parse(text = x))})
names(aamut.fisher) <- varlist


apply(aamut,2,function(e) fisher.test(table(factor(e,levels=c(0, 1)),
factor(metadata$Sex, levels=c(0, 1))))$p.value)

##    PB2_V255I    PB2_R299K    PB2_K340R    PB2_V480I    PB1_G216S
PB1_I517V
## 0.0112899779 0.2704129678 0.2199592195 0.0042945190 1.0000000000
1.0000000000
##     PA_I668V     HA_K92R     HA_N121K     HA_N122D     HA_T135K
HA_I140M
## 1.0000000000 0.1003346830 0.0498758159 0.3554995377 0.4147884077
1.0000000000
##     HA_R142K     HA_R142G     HA_S144K     HA_N171K     HA_R261Q
HA_S262N
## 0.7686657544 0.7215112733 0.0074841583 0.0059395383 0.4388632848
0.2484769756
##     HA_H311Q     HA_I406V     HA_G495E     HA_G484E     NP_V197I
NP_G450S
## 0.2199592195 0.0003625899 0.8819146930 0.0064332750 0.2634734129
0.0001968291
##     NP_T472A      NA_T19S      NA_S44P      NA_G93D     NA_L140I
NA_V143M
## 0.0049668463 0.2536716602 0.5954869639 0.0892471622 0.0596462887
0.4634843964
##     NA_V149A     NA_N161S     NA_I176M     NA_N329S     NA_D339N
NA_G346V
## 0.1089957903 0.0167920242 1.0000000000 0.7743481434 0.4634843964
0.3152945426
##     NA_P468L     NA_P468H     NS1_A56S     NS1_E71G     NS1_S99T
NS1_K108T
## 0.0184787483 0.0291357850 0.0303109462 0.0174375503 0.0042945190
1.0000000000
##    NS1_L146S    NS1_K196E
## 0.0023586687 0.2594836907

aamut.fun.cont2 <- (paste("apply(aamut, 2, function(e)table(factor(e,
levels=c(0,1)), factor(", varlist2,", levels=c(0, 1))))[,c(names(aamut))]",
sep=""))
aamut.fun.cont3 <- (paste("apply(aamut, 2, function(e)table(factor(e,
levels=c(0,1)), factor(", varlist3,", levels=c(0, 1,
2))))[,c(names(aamut))]", sep=""))
aamut.fun.cont<-c(aamut.fun.cont2,aamut.fun.cont3)
aamut.fisher.cont <- lapply(aamut.fun.cont, function(x) {eval(parse(text =
x))})
```

```
names(aamut.fisher.cont) <- varlist
aamut.fisher.cont2 <-list()
aamut.fisher.cont2 <- (melt(aamut.fisher.cont))
aamut.fisher.cont2$Name <-
paste(aamut.fisher.cont2$Var2,"_",aamut.fisher.cont2$L1,"_",aamut.fisher.cont
2$Var1, sep="")
rownames(aamut.fisher.cont2)<-aamut.fisher.cont2$Name
aamut.fisher.cont2$Var1<-NULL
aamut.fisher.cont2$Var2<-NULL
aamut.fisher.cont2$L1<-NULL
aamut.fisher.cont2$Name<-NULL

write.csv(x = aamut.fisher.cont2, file.path("aamut.fisher.cont2.csv"))


aamut.fun.fdr2 <- noquote(paste("p.adjust(apply(aamut,2,function(e)
fisher.test(table(factor(e,levels=c(0, 1)), factor(", varlist2,", levels=c(0,
1))))$p.value),method=\"fdr\")", sep=""))
aamut.fun.fdr3 <- noquote(paste("p.adjust(apply(aamut,2,function(e)
fisher.test(table(factor(e,levels=c(0, 1)), factor(", varlist3,", levels=c(0,
1, 2))))$p.value),method=\"fdr\")", sep=""))
aamut.fun.fdr <- c(aamut.fun.fdr2,aamut.fun.fdr3)
aamut.fisher.fdr <- lapply(aamut.fun.fdr, function(x) {eval(parse(text =
x))})
names(aamut.fisher.fdr) <- varlist


write.csv(aamut.fisher,file.path("Results/aamut.fisher.csv"))

write.csv(aamut.fisher.cont2,file.path("Results/aamut.fisher.cont.csv"))

write.csv(aamut.fisher.fdr,file.path("Results/aamut.fisher.fdr.csv"))



##A summary of the results of the file with the results from the fisher test
+ fdr (aamut.fisher.fdr) and from the numbers of samples belonging to each
group (aamut.fisher.cont2) is available in Supplementary File 2: "AAMut
Fisher + FDR"
```

Link between mutations

Here two models are constructed with all mutations that are significantly more present in women and all mutations that are significantly more present in men. This is to evaluate if there are (multi)collinearities between the mutations. The variance inflation factor (VIF) is checked to see how much variance of a coefficient is inflated because of linear dependence with other predictors. A value of 1 means that the predictor is not correlated with the other variables. The higher the value, the greater the correlation of the variable with the other variables. Values of more than 4 or 5 are regarded as moderate to high, with values of 10 or more being regarded as very high.

Sex

```
library(car)

modelmalemutations <- glm(Sex~ PB2_V255I + PB2_V480I + HA_S144K + NP_G450S +
NS1_S99T + NS1_L146S, data=metadataaamut, family="binomial")

## Warning: glm.fit: fitted probabilities numerically 0 or 1 occurred

summary(modelmalemutations)

##
## Call:
## glm(formula = Sex ~ PB2_V255I + PB2_V480I + HA_S144K + NP_G450S +
##     NS1_S99T + NS1_L146S, family = "binomial", data = metadataaamut)
##
## Deviance Residuals:
##    Min       1Q   Median       3Q      Max
## -1.291   -1.291    0.000    1.068    1.548
##
## Coefficients:
##                Estimate Std. Error z value Pr(>|z|)
## (Intercept)      0.2625     0.1481   1.772   0.0763 .
## PB2_V255I1      62.6777  3995.4272   0.016   0.9875
## PB2_V480I1     -15.9892  1577.0975  -0.010   0.9919
## HA_S144K1       18.3646  4436.9442   0.004   0.9967
## NP_G450S1      -47.2396  2915.8009  -0.016   0.9871
## NS1_S99T1      -50.0538  5040.5254  -0.010   0.9921
## NS1_L146S1      31.1381  2391.4800   0.013   0.9896
## ---
## Signif. codes:  0 '***' 0.001 '**' 0.01 '*' 0.05 '.' 0.1 ' ' 1
##
## (Dispersion parameter for binomial family taken to be 1)
##
##     Null deviance: 338.26  on 243  degrees of freedom
## Residual deviance: 311.13  on 237  degrees of freedom
##   (9 observations deleted due to missingness)
## AIC: 325.13
```

```
##
## Number of Fisher Scoring iterations: 17
```

```
modelfemalemutations <- glm(Sex~  NP_T472A + HA_N171K + HA_I406V + HA_G484E,
data=metadataaamut, family="binomial")
summary(modelfemalemutations)
```

```
##
## Call:
## glm(formula = Sex ~ NP_T472A + HA_N171K + HA_I406V + HA_G484E,
##     family = "binomial", data = metadataaamut)
##
## Deviance Residuals:
##     Min       1Q   Median       3Q      Max
## -1.7614  -1.2980   0.1593   1.0614   1.4846
##
## Coefficients:
##             Estimate Std. Error z value Pr(>|z|)
## (Intercept)  -0.6983     0.2754  -2.535   0.0112 *
## NP_T472A1     0.1154     0.6079   0.190   0.8494
## HA_N171K1    -0.9054     1.2069  -0.750   0.4532
## HA_I406V1     2.8013     1.3408   2.089   0.0367 *
## HA_G484E1    -1.0339     1.2029  -0.859   0.3901
## ---
## Signif. codes:  0 '***' 0.001 '**' 0.01 '*' 0.05 '.' 0.1 ' ' 1
##
## (Dispersion parameter for binomial family taken to be 1)
##
##     Null deviance: 338.26  on 243  degrees of freedom
## Residual deviance: 321.50  on 239  degrees of freedom
##   (9 observations deleted due to missingness)
## AIC: 331.5
##
## Number of Fisher Scoring iterations: 4
```

```
vif(modelmalemutations)
```

```
## PB2_V255I PB2_V480I  HA_S144K  NP_G450S  NS1_S99T NS1_L146S
## 127991186  19942093 157841630  68166243 203706662  45855044
```

```
vif(modelfemalemutations)
```

```
##  NP_T472A  HA_N171K  HA_I406V  HA_G484E
##  3.830922 15.838832 19.650117 15.931660
```

Sampling Period

```
library(car)

modelPeriod1mutations <- glm(Period~ HA_S144K + NA_G93D + NA_P468L +
NS1_L146S + NS1_S99T + PB2_V255I, data=metadataaamut, family="binomial")
summary(modelPeriod1mutations)
```

```
##
## Call:
## glm(formula = Period ~ HA_S144K + NA_G93D + NA_P468L + NS1_L146S +
##     NS1_S99T + PB2_V255I, family = "binomial", data = metadataaamut)
##
## Deviance Residuals:
##     Min       1Q   Median       3Q      Max
## -2.2833  -0.9889   0.8479   0.8479   1.1220
##
## Coefficients:
##             Estimate Std. Error z value Pr(>|z|)
## (Intercept)   0.8380     0.1565   5.354  8.6e-08 ***
## HA_S144K1     2.9911     1.6197   1.847   0.0648 .
## NA_G93D1      1.8560     1.8270   1.016   0.3097
## NA_P468L1    -0.3562     2.1748  -0.164   0.8699
## NS1_L146S1    0.5992     1.8800   0.319   0.7499
## NS1_S99T1    -1.3054     2.4198  -0.539   0.5895
## PB2_V255I1   -2.0927     2.4550  -0.852   0.3940
## ---
## Signif. codes:  0 '***' 0.001 '**' 0.01 '*' 0.05 '.' 0.1 ' ' 1
##
## (Dispersion parameter for binomial family taken to be 1)
##
##     Null deviance: 286.18  on 252  degrees of freedom
## Residual deviance: 270.05  on 246  degrees of freedom
## AIC: 284.05
##
## Number of Fisher Scoring iterations: 5
```

```
modelPeriod2mutations <- glm(Period~  PB1_G216S + PB1_I517V,
data=metadataaamut, family="binomial")
summary(modelPeriod2mutations)
```

```
##
## Call:
## glm(formula = Period ~ PB1_G216S + PB1_I517V, family = "binomial",
##     data = metadataaamut)
##
## Deviance Residuals:
##     Min       1Q   Median       3Q      Max
## -1.6815  -1.4592   0.7466   0.7466   0.9196
##
## Coefficients:
##              Estimate Std. Error z value Pr(>|z|)
## (Intercept)    1.1350     0.1564   7.255    4e-13 ***
## PB1_G216S1   -14.9242  1029.1215  -0.015    0.988
## PB1_I517V1    14.4311  1029.1215   0.014    0.989
## ---
## Signif. codes:  0 '***' 0.001 '**' 0.01 '*' 0.05 '.' 0.1 ' ' 1
##
```

```
## (Dispersion parameter for binomial family taken to be 1)
##
##     Null deviance: 286.18  on 252  degrees of freedom
## Residual deviance: 283.69  on 250  degrees of freedom
## AIC: 289.69
##
## Number of Fisher Scoring iterations: 14
```

```r
vif(modelPeriod1mutations)
```

```
##  HA_S144K   NA_G93D  NA_P468L NS1_L146S  NS1_S99T PB2_V255I
##  8.794774 12.531605 17.569936 12.952511 21.611807 22.364366
```

```r
vif(modelPeriod2mutations)
```

```
## PB1_G216S PB1_I517V
##   5980103   5980103
```

Logistic regression

Host characteristics

```
logreglist <- c("Sex","Period")

varlist <- c("Surveillance", "Sex", "ca_vacci", "ca_status", "ca_antibio",
"asthma_respi", "cardio", "obesity", "diabete", "renal", "immuno", "neuro",
"hepato", "icuever","Period", "Age.cat", "MildModerateSevere")

combo <- paste(outer(logreglist,varlist,paste, sep=" ~ "),sep="")

modelscharcomp <- lapply(combo, function(x) {
  glm(x, family=binomial, data = metadata)})

names(modelscharcomp) <- combo

##lapply(modelscharcomp, summary)
charcompvector2 <- sapply(modelscharcomp,function(f)
summary(f)$coefficients[,'Pr(>|z|)'][2])
charcompvector32 <- sapply(modelscharcomp,function(f)
summary(f)$coefficients[,'Pr(>|z|)'][2])
charcompvector33 <- sapply(modelscharcomp,function(f)
summary(f)$coefficients[,'Pr(>|z|)'][3])

charcompvector <- cbind(combo,
charcompvector2,charcompvector32,charcompvector33)

write.csv(charcompvector,file.path("Logistic Regression & Effect
Size","charcompvector.csv"))



##A summary of the results of this file is available in Supplementary File 2:
“Characteristics”
```

Confounding factors and effect modification

It could be that there exists confounding and/or interactions with other variables.

Confounding: A distortion of an estimated association caused by an unequal distribution of another risk factor. Solution: Add the potential confounding risk factor to the model (as a predictor) and see if the parameter estimate for your predictor of interest changes by more than 10% from the unadjusted, or crude, estimate.

Effect modification: To identify whether the effect of the exposure (i.e. risk factor) is different in groups of patients with different characteristics. In the presence of significant effect modification (or interaction), it is inappropriate to present pooled results. Instead, the goal should to describe effect modification and present results that are stratified.

The possible conclusions could be:

- **If there is only confounding:** The stratum-specific measures of association will be similar to one another, but they will be different from the overall crude estimate by 10% or more.
- **If there is neither confounding nor effect modification:** The crude estimate of association and the stratum-specific estimates will be similar. They don't have to be identical, just similar.
- **If there is only effect modification:** The stratum-specific estimates will differ from one another significantly.
- **If there is both effect modification and confounding:** You need to consider two possibilities:
  - If the stratum-specific estimates differ from one another, and they are both less than the crude estimate or if they are both greater than the crude estimate, then there is both confounding and effect modification.
  - If the stratum-specific estimates differ from one another, and the crude estimate is between the two stratum-specific estimates, then you need to pool the stratum-specific estimates (with a Mantel-Haenszel equation) to determine whether the pooled estimate is more than 10% different from the crude estimate.

```
##A summary of the results of these files (confounders and effect size) is
available in Supplementary File 2: “Effect Size”
```

Sex

```
mutSexlist <- c("PB2_V255I", "PB2_V480I", "HA_S144K", "HA_N171K", "HA_I406V",
"HA_G484E", "NP_G450S", "NP_T472A", "NS1_S99T", "NS1_L146S")

varlist <- c("Surveillance", "Sex", "ca_vacci", "ca_status", "ca_antibio",
"asthma_respi", "cardio", "obesity", "diabete", "renal", "immuno", "neuro",
"hepato", "icuever","Period", "Age.cat", "MildModerateSevere")

Sexcombo2cf <- outer(mutSexlist,varlist,paste, sep=" + ")
Sexcombo20cf <- paste("Sex ~ ",Sexcombo2cf,sep = "")
Sexmodel2cf <- lapply(Sexcombo20cf, function(x) {glm(x, family=binomial, data
```

```
= metadataaamut)})


##lapply(Sexmodel2cf, summary)
Sexconfoundingfactor2cf <- sapply(Sexmodel2cf,function(f)
summary(f)$coefficients[2])

Sexcombo2em <- outer(mutSexlist,varlist,paste, sep=" * ")
Sexcombo20em <- paste("Sex ~ ",Sexcombo2em,sep = "")
Sexmodel2em <- lapply(Sexcombo20em, function(x) {glm(x, family=binomial, data
= metadataaamut)})



#lapply(Sexmodel2em, summary)
Sexestimate2emest <- sapply(Sexmodel2em,function(f)
summary(f)$coefficients[2])
Sexestimate2empval2 <- sapply(Sexmodel2em,function(f)
summary(f)$coefficients[,'Pr(>|z|)'][4])
Sexestimate2empval32 <- sapply(Sexmodel2em,function(f)
summary(f)$coefficients[,'Pr(>|z|)'][5])
Sexestimate2empval33 <- sapply(Sexmodel2em,function(f)
summary(f)$coefficients[,'Pr(>|z|)'][6])

vectorSex <-
cbind(Sexcombo20cf,Sexconfoundingfactor2cf,Sexestimate2emest,Sexestimate2empv
al2,Sexestimate2empval32,Sexestimate2empval33)

write.csv(vectorSex,file.path("Logistic Regression & Effect
Size","LogReg_SexMut.csv"))
```

Sampling Period

```
mutPeriodlist <- c("PB2_V255I", "PB1_G216S", "PB1_I517V", "HA_S144K",
"NA_G93D", "NA_P468L", "NA_P468H", "NS1_S99T", "NS1_L146S")

varlist <- c("Surveillance", "Sex", "ca_vacci", "ca_status", "ca_antibio",
"asthma_respi", "cardio", "obesity", "diabete", "renal", "immuno", "neuro",
"hepato", "icuever","Period", "Age.cat", "MildModerateSevere")

Periodcombo2cf <- outer(mutPeriodlist,varlist,paste, sep=" + ")
Periodcombo20cf <- paste("Period ~ ",Periodcombo2cf,sep = "")
Periodmodel2cf <- lapply(Periodcombo20cf, function(x) {glm(x,
family=binomial, data = metadataaamut)})

##lapply(Periodmodel2cf, summary)
Periodconfoundingfactor2cf <- sapply(Periodmodel2cf,function(f)
summary(f)$coefficients[2])

Periodcombo2em <- outer(mutPeriodlist,varlist,paste, sep=" * ")
Periodcombo20em <- paste("Period ~ ",Periodcombo2em,sep = "")
```

```
Periodmodel2em <- lapply(Periodcombo20em, function(x) {glm(x,
family=binomial, data = metadataaamut)})



#lapply(Periodmodel2em, summary)
Periodestimate2emest <- sapply(Periodmodel2em,function(f)
summary(f)$coefficients[2])
Periodestimate2empval2 <- sapply(Periodmodel2em,function(f)
summary(f)$coefficients[,'Pr(>|z|)'][4])
Periodestimate2empval32 <- sapply(Periodmodel2em,function(f)
summary(f)$coefficients[,'Pr(>|z|)'][5])
Periodestimate2empval33 <- sapply(Periodmodel2em,function(f)
summary(f)$coefficients[,'Pr(>|z|)'][6])

vectorPeriod <-
cbind(Periodcombo20cf,Periodconfoundingfactor2cf,Periodestimate2emest,Periode
stimate2empval2,Periodestimate2empval32,Periodestimate2empval33)

write.csv(vectorPeriod,file.path("Logistic Regression & Effect
Size","LogReg_PeriodMut.csv"))
```

Effect size

```
##A summary of the results of these files (confounders and effect size) is
available in Supplementary File 2: “Effect Size”

mutlist <- c("PB2_V255I", "PB2_V480I", "HA_S144K", "HA_N171K", "HA_I406V",
"HA_G484E", "NP_G450S", "NP_T472A", "NS1_S99T", "NS1_L146S", "PB1_I517V",
"PB2_V255I", "PB1_G216S", "PB1_I517V", "HA_S144K", "NA_G93D", "NA_P468L",
"NA_P468H", "NS1_S99T", "NS1_L146S")

metadataaamut$Period <- factor(metadataaamut$Period, levels = c(0,1,2))

charSexlist <- rep("Sex", times = 10)
charPeriodlist <- rep("Period", times = 9)
charlist1 <- c(charSexlist, charPeriodlist)
comboeffectsize <- paste(mutlist,charlist1,sep = " ~ ")

modeleffectsize1 <- lapply(comboeffectsize, function(x) {glm(x,
family=binomial, data = metadataaamut)})
#lapply(modeleffectsize, summary)
expeffectsize1 <- lapply(modeleffectsize1, function(x)
{exp(cbind(coef(x),confint(x)))})

metadataaamut$Period <- factor(metadataaamut$Period, levels = c(1,2,0))
modeleffectsize2 <- lapply(comboeffectsize, function(x) {glm(x,
family=binomial, data = metadataaamut)})
#lapply(modeleffectsize, summary)
```

```
expeffectsize2 <- lapply(modeleffectsize2, function(x)
{exp(cbind(coef(x),confint(x)))})

effectsize1oddsratio_2 <- rep("NA",length(charlist1))
effectsize1oddsratio_2[1:10] <- sapply(expeffectsize1[1:10],function(f)
f[2,1])
effectsize1oddsCI2.5_2 <- rep("NA",length(charlist1))
effectsize1oddsCI2.5_2[1: 10] <- sapply(expeffectsize1[1:10],function(f)
f[2,2])
effectsize1oddsCI97.5_2 <- rep("NA",length(charlist1))
effectsize1oddsCI97.5_2[1: 10] <- sapply(expeffectsize1[1:10],function(f)
f[2,3])

effectsize1oddsratio_32 <- rep("NA",length(charlist1))
effectsize1oddsratio_32[11:19] <- sapply(expeffectsize1[11:19],function(f)
f[2,1])
effectsize1oddsCI2.5_32 <- rep("NA",length(charlist1))
effectsize1oddsCI2.5_32[11:19] <- sapply(expeffectsize1[11:19],function(f)
f[2,2])
effectsize1oddsCI97.5_32 <- rep("NA",length(charlist1))
effectsize1oddsCI97.5_32[11:19] <- sapply(expeffectsize1[11:19],function(f)
f[2,3])
effectsize1oddsratio_33 <- rep("NA",length(charlist1))
effectsize1oddsratio_33[11:19] <- sapply(expeffectsize1[11:19],function(f)
f[3,1])
effectsize1oddsCI2.5_33 <- rep("NA",length(charlist1))
effectsize1oddsCI2.5_33[11:19] <- sapply(expeffectsize1[11:19],function(f)
f[3,2])
effectsize1oddsCI97.5_33 <- rep("NA",length(charlist1))
effectsize1oddsCI97.5_33[11:19] <- sapply(expeffectsize1[11:19],function(f)
f[3,3])
effectsize2oddsratio_32 <- rep("NA",length(charlist1))
effectsize2oddsratio_32[11:19] <- sapply(expeffectsize2[11:19],function(f)
f[2,1])
effectsize2oddsCI2.5_32 <- rep("NA",length(charlist1))
effectsize2oddsCI2.5_32[11:19] <- sapply(expeffectsize2[11:19],function(f)
f[2,2])
effectsize2oddsCI97.5_32 <- rep("NA",length(charlist1))
effectsize2oddsCI97.5_32[11:19] <- sapply(expeffectsize2[11:19],function(f)
f[2,3])
effectsize2oddsratio_33 <- rep("NA",length(charlist1))
effectsize2oddsratio_33[11:19] <- sapply(expeffectsize2[11:19],function(f)
f[3,1])
effectsize2oddsCI2.5_33 <- rep("NA",length(charlist1))
effectsize2oddsCI2.5_33[11:19] <- sapply(expeffectsize2[11:19],function(f)
f[3,2])
effectsize2oddsCI97.5_33 <- rep("NA",length(charlist1))
effectsize2oddsCI97.5_33[11:19] <- sapply(expeffectsize2[11:19],function(f)
f[3,3])
```

```
vectoreffectsize <-
cbind(comboeffectsize,effectsize1oddsratio_2,effectsize1oddsCI2.5_2,effectsiz
e1oddsCI97.5_2,effectsize1oddsratio_32,effectsize1oddsCI2.5_32,effectsize1odd
sCI97.5_32,effectsize1oddsratio_33,effectsize1oddsCI2.5_33,effectsize1oddsCI9
7.5_33,effectsize2oddsratio_32,effectsize2oddsCI2.5_32,effectsize2oddsCI97.5_
32,effectsize2oddsratio_33,effectsize2oddsCI2.5_33,effectsize2oddsCI97.5_33)


write.csv(vectoreffectsize,file.path("Logistic Regression & Effect
Size","EffectSize.csv"))
```

Are mutations within phylogenetic groups linked to specific host characteristics

```r
library(adegenet)
library(glmnet)
library(ShortRead)
library("DECIPHER")
library(msa)
library("FactoMineR")
library("factoextra")
library(rcompanion)
library(RVAideMemoire)
library(fmsb)
library(reshape2)
library(plyr)
library(dplyr)
library(janitor)
library(stringr)
library(ggpubr)
library(purrr)
library(ggplot2)
library(tidyr)
```

Read in data

Metadata

- 160 **SARI** samples
- 93 **ILI** samples

```
metadata= read_excel("2. Supplementary File - Input files and results
statistical test.xlsx", sheet = "METADATA", header = TRUE, sep = ",", quote =
"\"", dec = ".")
# n=253

#ILI=0; SARI=1
#Male=0; Female=1
#No=0; Yes=1
#Alive=0; Deceased=1
#Beginning=0; Middle=1; End=2
#<15=0; 15-59=1; >=60=2
#Mild=0; Moderate=1; Severe=2


metadata$Reference <- (as.character(metadata$Reference))

metadata[metadata == "16-IG0682"] <- "16-IG-0682"
metadata[metadata == "16-IG0711"] <- "16-IG-0711"
metadata[metadata == "16-IG0712"] <- "16-IG-0712"
metadata[metadata == "16-IG0720"] <- "16-IG-0720"

metadata[] <- lapply(metadata,factor)

groups= read_excel("2. Supplementary File - Input files and results
statistical test.xlsx", sheet = "WG", header = TRUE, sep = ",", quote = "\"",
dec = ".")

groups[] <- lapply(groups,factor)

metadata<- metadata[match(groups$X, as.character(metadata$Reference)),]
metadatagroups <- cbind(metadata,groups)

metadata3C2a<-metadatagroups[metadatagroups$Group.3C.2a==1,]
#3C 2a 1 and 3C 2a 1 (2) are genetically still very close in comparison to
the other groups, so to increase the sample size and the power of the
analysis, these two groups are taken together
metadata3C2a1<-metadatagroups[metadatagroups$Group.3C.2a.1==1,]
metadata3C2a1_2<-metadatagroups[metadatagroups$Group.3C.2a.1..2.==1,]
metadata3C2a1_total <- rbind(metadata3C2a1, metadata3C2a1_2)
#3C 2a 1a and 3C 2a 1a (2) are genetically still very close in comparison to
the other groups, so to increase the sample size and the power of the
analysis, these two groups are taken together
metadata3C2a1a<-metadatagroups[metadatagroups$Group.3C.2a.1a==1,]
```

```
metadata3C2a1a_2<-metadatagroups[metadatagroups$Group.3C.2a.1a..2.==1,]
metadata3C2a1a_total <- rbind(metadata3C2a1a, metadata3C2a1a_2)

metadata3C2a1b<-metadatagroups[metadatagroups$Group.3C.2a.1b==1,]
metadata3C2a2<-metadatagroups[metadatagroups$Group.3C.2a.2==1,]
metadataGroup1<-metadatagroups[metadatagroups$Group.1==1,]
metadata3C2a3<-metadatagroups[metadatagroups$Group.3C.2a.3==1,]

metadataWG.1<-metadatagroups[metadatagroups$WG.1==1,]
metadataWG.2<-metadatagroups[metadatagroups$WG.2==1,]


metadataPHYLOGROUPX <-
rbind(metadata3C2a1_total,metadata3C2a1a_total,metadata3C2a1b,metadata3C2a2,m
etadataGroup1,metadataWG.1, metadataWG.2)
```

List of mutations

Subset: prioritized mutations: *amino acids*

- Non-synonymous amino acids
- Filtering frequency 5-95%
- Coverage >100
- Biallelic and triallelic –> triallelic: added extra variable

Subset 1: amino acids

```
aamut= read_excel("2. Supplementary File - Input files and results
statistical test.xlsx", sheet = "Non-Synonymous aa", header = F, sep = ",",
quote = "\"", stringsAsFactors=F)

aamut=t(aamut)
colnames(aamut)=aamut[1,]
rownames(aamut)=aamut[,1]
aamut=aamut[-1,-1]
aamut=as.data.frame(aamut)

# Coverage already checked --> ok
# all aa mutations coverage of >100

dim(aamut) # 42 amino acid mutations

## [1] 253  44

aamut[] <- lapply(aamut,factor)


aamut$Reference <- rownames(aamut)
```

```
aamut3C2a <- (aamut[rownames(aamut) %in% metadata3C2a$Reference,])
aamut3C2a$Reference <- NULL
metadataaamut3C2a <- merge(metadata3C2a,aamut,by=c("Reference"))


aamut3C2a3 <- (aamut[rownames(aamut) %in% metadata3C2a3$Reference,])
aamut3C2a3$Reference <- NULL
metadataaamut3C2a3<-merge(metadata3C2a3,aamut,by=c("Reference"))

aamutPHYLOGROUPX <- (aamut[rownames(aamut) %in%
metadataPHYLOGROUPX$Reference,])
aamutPHYLOGROUPX$Reference <- NULL
metadataaamutPHYLOGROUPX<-merge(metadataPHYLOGROUPX,aamut,by=c("Reference"))
```

Univariate analysis: GWAS

```
GWAS <- function(metadatafilename,mutationfilename) {

varlist2 <- c(paste(substitute(metadatafilename),"$Surveillance",sep=""),
paste(substitute(metadatafilename),"$MildModerateVSSevere",sep=""),
paste(substitute(metadatafilename),"$Sex",sep=""),
paste(substitute(metadatafilename),"$ca_vacci",sep=""),
paste(substitute(metadatafilename),"$ca_status",sep=""),
paste(substitute(metadatafilename),"$ca_antibio",sep=""),
paste(substitute(metadatafilename),"$asthma_respi",sep=""),
paste(substitute(metadatafilename),"$cardio",sep=""),
paste(substitute(metadatafilename),"$obesity",sep=""),
paste(substitute(metadatafilename),"$diabete",sep=""),
paste(substitute(metadatafilename),"$renal",sep=""),
paste(substitute(metadatafilename),"$immuno",sep=""),
paste(substitute(metadatafilename),"$neuro",sep=""),
paste(substitute(metadatafilename),"$hepato",sep=""),
paste(substitute(metadatafilename),"$icuever",sep=""))

varlist3 <- c(paste(substitute(metadatafilename),"$Period",sep=""),
paste(substitute(metadatafilename),"$Age.cat",sep=""),
paste(substitute(metadatafilename),"$MildModerateSevere",sep=""))

#, paste(substitute(metadatafilename),"$ModeratevsSevere",sep=""),
paste(substitute(metadatafilename),"$MildvsSevere",sep="")
#,"ModeratevsSevere","MildvsSevere"

varlist <- c("Surveillance","MildModerateVSSevere", "Sex", "ca_vacci",
"ca_status", "ca_antibio", "asthma_respi", "cardio", "obesity", "diabete",
"renal", "immuno", "neuro", "hepato", "icuever","Period", "Age.cat",
"MildModerateSevere")

fisher <- list()
fun2 <- (paste("apply(",substitute(mutationfilename),",2,function(e)
fisher.test(table(factor(e,levels=c(0, 1)), factor(", varlist2,", levels=c(0,
```

```
1))))$p.value)", sep=""))
fun3 <- (paste("apply(",substitute(mutationfilename),",2,function(e)
fisher.test(table(factor(e,levels=c(0, 1)), factor(", varlist3,", levels=c(0,
1, 2))))$p.value)", sep=""))
fun <- c(fun2,fun3)
fisher <- lapply(fun, function(x) {eval(parse(text = x))})
names(fisher) <- varlist

fun.cont2 <- (paste("apply(",substitute(mutationfilename),", 2,
function(e)table(factor(e, levels=c(0,1)), factor(", varlist2,", levels=c(0,
1))))[,c(names(",substitute(mutationfilename),"))]", sep=""))
fun.cont3 <- (paste("apply(",substitute(mutationfilename),", 2,
function(e)table(factor(e, levels=c(0,1)), factor(", varlist3,", levels=c(0,
1, 2))))[,c(names(",substitute(mutationfilename),"))]", sep=""))
fun.cont<-c(fun.cont2,fun.cont3)

fisher.cont <- list()
fisher.cont <- lapply(fun.cont, function(x) {eval(parse(text = x))})
names(fisher.cont) <- varlist
fisher.cont2 <-list()
fisher.cont2 <- (melt(fisher.cont))
fisher.cont2$Name <-
paste(fisher.cont2$Var2,"_",fisher.cont2$L1,"_",fisher.cont2$Var1, sep="")
rownames(fisher.cont2)<-fisher.cont2$Name
fisher.cont2$Var1<-NULL
fisher.cont2$Var2<-NULL
fisher.cont2$L1<-NULL
fisher.cont2$Name<-NULL

fisher.fdr <- list()
fun.fdr2 <-
noquote(paste("p.adjust(apply(",substitute(mutationfilename),",2,function(e)
fisher.test(table(factor(e,levels=c(0, 1)), factor(", varlist2,", levels=c(0,
1))))$p.value),method=\"fdr\")", sep=""))
fun.fdr3 <-
noquote(paste("p.adjust(apply(",substitute(mutationfilename),",2,function(e)
fisher.test(table(factor(e,levels=c(0, 1)), factor(", varlist3,", levels=c(0,
1, 2))))$p.value),method=\"fdr\")", sep=""))
fun.fdr <- c(fun.fdr2,fun.fdr3)
fisher.fdr <- lapply(fun.fdr, function(x) {eval(parse(text = x))})
names(fisher.fdr) <- varlist

Resultsfisher <-
paste("Results/fisher_",substitute(mutationfilename),".csv",sep="")

write.csv(fisher,file.path(Resultsfisher))

Resultsfisher.cont <-
paste("Results/fisher.cont_",substitute(mutationfilename),".csv",sep="")
```

```
write.csv(fisher.cont2,file.path(Resultsfisher.cont))

Resultsfisher.fdr <-
paste("Results/fisher.fdr_",substitute(mutationfilename),".csv",sep="")

write.csv(fisher.fdr,file.path(Resultsfisher.fdr))
}
#Amino acids
GWAS(metadataaamut3C2a,aamut3C2a)
GWAS(metadataaamut3C2a3,aamut3C2a3)
GWAS(metadataaamutPHYLOGROUPX,aamutPHYLOGROUPX)



##A summary of these files (fisher.cont2, fisher.fdr) are saved in
Supplementary File 2 as “AAMut Fisher + FDR (3C2a)”, “AAMut Fisher + FDR
(3C2a3)” and “AAMut Fisher + FDR (PHYLOGROUP 1)”
```

Link between mutations

It seems that some mutations seem to occur together in the same samples. We evaluate if there are (multi)collinearities between the mutations. The variance inflation factor (VIF) is checked to see how much variance of a coefficient is inflated because of linear dependence with other predictors. A value of 1 means that the predictor is not correlated with the other variables. The higher the value, the greater the correlation of the variable with the other variables. Values of more than 4 or 5 are regarded as moderate to high, with values of 10 or more being regarded as very high.

```
library(car)

#HA_H311Q and HA_K92R are in all the same samples
aamut_PHYLOGROUPX_vif_1 <- glm(renal ~ PB2_R299K+PB2_K340R+HA_K92R+NP_V197I,
data = metadataaamutPHYLOGROUPX, family = "binomial")
summary(aamut_PHYLOGROUPX_vif_1)

##
## Call:
## glm(formula = renal ~ PB2_R299K + PB2_K340R + HA_K92R + NP_V197I,
##     family = "binomial", data = metadataaamutPHYLOGROUPX)
##
## Deviance Residuals:
##     Min       1Q   Median       3Q      Max
## -1.1774  -0.4265  -0.4265  -0.4265   2.2101
##
## Coefficients:
##             Estimate Std. Error z value Pr(>|z|)
## (Intercept)  -1.3863     1.1180  -1.240    0.215
## PB2_R299K1   -0.9651     1.1525  -0.837    0.402
## PB2_K340R1   -3.1623     2.2998  -1.375    0.169
```

```
## HA_K92R1      2.3514     1.4416   1.631    0.103
## NP_V197I1      1.6582     1.2563   1.320    0.187
##
## (Dispersion parameter for binomial family taken to be 1)
##
##     Null deviance: 144.14  on 189  degrees of freedom
## Residual deviance: 131.74  on 185  degrees of freedom
## AIC: 141.74
##
## Number of Fisher Scoring iterations: 5
```

```
vif(aamut_PHYLOGROUPX_vif_1)
```

```
## PB2_R299K PB2_K340R  HA_K92R  NP_V197I
##  5.046077 17.990776 7.601777  5.898579
```

Logistic regression

Host characteristics

```
logreglist <- c("renal")

varlist <- c("Surveillance", "Sex", "ca_vacci", "ca_status", "ca_antibio",
"asthma_respi", "cardio", "obesity", "diabete", "renal", "immuno", "neuro",
"hepato", "icuever","Period", "Age.cat", "MildModerateSevere")
metadatafiles <- c("metadataaamutPHYLOGROUPX")

combo <- paste(outer(logreglist,varlist,paste, sep=" ~ "),sep="")
combo <- combo[!combo %in% "renal ~ renal"]

models <- NULL
models <- (paste("lapply(\"",combo,"\",function(x) { glm(x, family =
binomial, data = ", sep=""))
models <- (paste(outer(models,metadatafiles,paste,sep=""), sep=""))
models <- paste(models, ")})", sep="")

modelscharcomp <- list()
modelscharcomp <- lapply(models, function(x) {eval(parse(text = x))})
modelscharcomp <- map(modelscharcomp,1)
names(modelscharcomp) <- models
#lapply(modelscharcomp, summary)

charcompvector2 <- sapply(modelscharcomp,function(f)
summary(f)$coefficients[,'Pr(>|z|)'][2])
charcompvector32 <- sapply(modelscharcomp,function(f)
summary(f)$coefficients[,'Pr(>|z|)'][2])
charcompvector33 <- sapply(modelscharcomp,function(f)
summary(f)$coefficients[,'Pr(>|z|)'][3])
```

```
charcompvector <- cbind( charcompvector2,charcompvector32,charcompvector33)

write.csv(charcompvector,file.path("LogReg &
ES/Results","charcompvector.csv"))

##A summary of this files is saved in Supplementary File 2 as
“Characteristics (Phylo)”
```

Confounding factors and effect modification

It could be that there exists confounding and/or interactions with other variables.

Confounding: A distortion of an estimated association caused by an unequal distribution of another risk factor. Solution: Add the potential confounding risk factor to the model (as a predictor) and see if the parameter estimate for your predictor of interest changes by more than 10% from the unadjusted, or crude, estimate.

Effect modification: To identify whether the effect of the exposure (i.e. risk factor) is different in groups of patients with different characteristics. In the presence of significant effect modification (or interaction), it is inappropriate to present pooled results. Instead, the goal should to describe effect modification and present results that are stratified.

The possible conclusions could be:

- **If there is only confounding:** The stratum-specific measures of association will be similar to one another, but they will be different from the overall crude estimate by 10% or more.
- **If there is neither confounding nor effect modification:** The crude estimate of association and the stratum-specific estimates will be similar. They don’t have to be identical, just similar.
- **If there is only effect modification:** The stratum-specific estimates will differ from one another significantly.
- **If there is both effect modification and confounding:** You need to consider two possibilities:
  - If the stratum-specific estimates differ from one another, and they are both less than the crude estimate or if they are both greater than the crude estimate, then there is both confounding and effect modification.
  - If the stratum-specific estimates differ from one another, and the crude estimate is between the two stratum-specific estimates, then you need to pool the stratum-specific estimates (with a Mantel-Haenszel equation) to determine whether the pooled estimate is more than 10% different from the crude estimate.

```
##A summary of these files (confounders and effect size) are saved in
Supplementary File 2 as “Effect Size (Phylo)”
```

```
mutlistPHYLOGROUPX <- c("PB2_R299K", "PB2_K340R", "HA_K92R","HA_H311Q",
"NP_V197I")
signvarlistPHYLOGROUPX <- c("renal")

varlist <- c("Surveillance", "Sex", "ca_vacci", "ca_status", "ca_antibio",
"asthma_respi", "cardio", "obesity", "diabete", "renal", "immuno", "neuro",
"hepato", "icuever","Period", "Age.cat", "MildModerateSevere")

#confounding factor
combocfPHYLOGROUPX <- outer(mutlistPHYLOGROUPX,varlist,paste, sep=" + ")

combocf2PHYLOGROUPX <-
unlist(list(outer(signvarlistPHYLOGROUPX,combocfPHYLOGROUPX,paste, sep=" ~
")))

combocf3PHYLOGROUPX <- paste("lapply(\"",combocf2PHYLOGROUPX,"\",function(x)
{ glm(x, family = binomial, data = metadataaamutPHYLOGROUPX)})", sep="")

combocf <- c(combocf3PHYLOGROUPX)
modelcf <- lapply(combocf, function(x) {eval(parse(text = x))})

modelcf <- map(modelcf,1)
names(modelcf) <- combocf

#lapply(modelcf, summary)
modelconfoundingfactor <- sapply(modelcf, function(f)
summary(f)$coefficients[2])

#effect modification
comboemPHYLOGROUPX <- outer(mutlistPHYLOGROUPX,varlist,paste, sep=" * ")

comboem2PHYLOGROUPX <-
unlist(list(outer(signvarlistPHYLOGROUPX,comboemPHYLOGROUPX,paste, sep=" ~
")))

comboem3PHYLOGROUPX <- paste("lapply(\"",comboem2PHYLOGROUPX,"\",function(x)
{ glm(x, family = binomial, data = metadataaamutPHYLOGROUPX)})", sep="")

comboem <- c(comboem3PHYLOGROUPX)
modelem <- lapply(comboem, function(x) {eval(parse(text = x))})

modelem <- map(modelem,1)
names(modelem) <- comboem

modeleffectmodification <- sapply(modelem, function(f)
summary(f)$coefficients[2])

#lapply(modelem, summary)
```

```
modelestimate2emest <- sapply(modelem,function(f) summary(f)$coefficients[2])
modelestimate2empval2 <- sapply(modelem,function(f)
summary(f)$coefficients[,'Pr(>|z|)'][4])
modelestimate2empval32 <- sapply(modelem,function(f)
summary(f)$coefficients[,'Pr(>|z|)'][5])
modelestimate2empval33 <- sapply(modelem,function(f)
summary(f)$coefficients[,'Pr(>|z|)'][6])

vectormodel <-
cbind(combocf,modelconfoundingfactor,modelestimate2emest,modelestimate2empval
2,modelestimate2empval32,modelestimate2empval33)

write.csv(vectormodel,file.path("LogReg & ES/Results","LogReg.csv"))
```

Effect size

```
##A summary of these files (confounders and effect size) are saved in
Supplementary File 2 as "Effect Size (Phylo)"
```

```
mutlistPHYLOGROUPX <- c("PB2_R299K", "PB2_K340R", "HA_K92R","HA_H311Q",
"NP_V197I")
signvarlistPHYLOGROUPX <- c("renal")

metadataaamutPHYLOGROUPX[which(grepl("17-IG-
00020",metadataaamutPHYLOGROUPX$Reference)),which(colnames(metadataaamutPHYLO
GROUPX)=="PB2_R299K")] <- 0
metadataaamutPHYLOGROUPX[which(grepl("17-IG-
00020",metadataaamutPHYLOGROUPX$Reference)),which(colnames(metadataaamutPHYLO
GROUPX)=="NS1_A56S")] <- 0

comboef2aamutPHYLOGROUPX <-
unlist(list(outer(mutlistPHYLOGROUPX,signvarlistPHYLOGROUPX,paste, sep=" ~
")))

comboef3aamutPHYLOGROUPX <-
paste("lapply(\"",comboef2aamutPHYLOGROUPX,"\",function(x) { glm(x, family =
binomial, data = metadataaamutPHYLOGROUPX)})", sep="")

comboef <- c(comboef3aamutPHYLOGROUPX)

greprenal <- grep("renal", comboef, value=TRUE)
comboef2 <- c(greprenal)

modelef1 <- lapply(comboef2, function(x) {tryCatch(eval(parse(text =
x)),error = function(e) NULL)})
modelef1 <- map(modelef1,1)
#names(modelef1) <- comboef2
```

```
expeffectsize1 <- lapply(modelef1, function(x)
{tryCatch(exp(cbind(coef(x),confint(x))), error = function(e) NULL)})

#lapply(modelef3[[1]], summary)

effectsize1oddsratio_2 <- rep("NA",length(comboef))
effectsize1oddsratio_2 <- sapply(expeffectsize1,function(f) f[2,1])
effectsize1oddsCI2.5_2 <- rep("NA",length(comboef))
effectsize1oddsCI2.5_2 <- sapply(expeffectsize1,function(f) f[2,2])
effectsize1oddsCI97.5_2 <- rep("NA",length(comboef))
effectsize1oddsCI97.5_2 <- sapply(expeffectsize1,function(f) f[2,3])

vectoreffectsize <-
cbind(effectsize1oddsratio_2,effectsize1oddsCI2.5_2,effectsize1oddsCI97.5_2)
rownames(vectoreffectsize) <- c(comboef2)


write.csv(vectoreffectsize,file.path("LogReg & ES/Results","EffectSize.csv"))
```

Comparison to GISAID samples

```
library(adegenet)
library(glmnet)
library(ShortRead)
library("DECIPHER")
library(msa)
library("FactoMineR")
library("factoextra")
library(rcompanion)
library(RVAideMemoire)
library(fmsb)
library(adegenet)
library(glmnet)
library(ShortRead)
library("DECIPHER")
library(msa)
library("FactoMineR")
library("factoextra")
library(stats)
library(ade4)
library(ape)
library("Biostrings")
library("ggplot2")
library("ggtree")
library("cluster")
library(seqinr)
library(matrixStats)
library(ggplot2)
library("lattice")
library(miscTools)
library(reshape2)
library(dplyr)
library(janitor)
library(stringr)
library(ggpubr)
library(purrr)
library(readxl)
```

Global context: Read in data

Metadata

Original dataset (selected for sequencing)

```
metadata <- read_excel("2. Supplementary File - Input files and results
statistical test.xlsx", sheet = "GISAID_Samples")
```

```
metadataphylox <- metadata[which(metadata$Groups=="Phylogroup 1"),]
metadata3C2a3 <- metadata[which(metadata$Groups=="Group 3C 2a 3"),]
```

Sequences

```
PB2seq <- readDNAStringSet("H3N2_PB2_seq.fasta")
length(PB2seq)

## [1] 14157

dfPB2 <- NULL
dfPB2$Isolate_Name <- as.character(names(PB2seq))
dfPB2$Isolate_Name <- gsub("\\|(PB2).*","",dfPB2$Isolate_Name)
dfPB2$PB2seq <- paste(PB2seq)

PB1seq <- readDNAStringSet("H3N2_PB1_seq.fasta")
length(PB1seq)

## [1] 14157

dfPB1 <- NULL
dfPB1$Isolate_Name <- as.character(names(PB1seq))
dfPB1$Isolate_Name <- gsub("\\|(PB1).*","",dfPB1$Isolate_Name)
dfPB1$PB1seq <- paste(PB1seq)

PAseq <- readDNAStringSet("H3N2_PA_seq.fasta")
length(PAseq)

## [1] 14157

dfPA <- NULL
dfPA$Isolate_Name <- as.character(names(PAseq))
dfPA$Isolate_Name <- gsub("\\|(PA).*","",dfPA$Isolate_Name)
dfPA$PAseq <- paste(PAseq)

HAseq <- readDNAStringSet("H3N2_HA_seq.fasta")
length(HAseq)

## [1] 14157

dfHA <- NULL
dfHA$Isolate_Name <- as.character(names(HAseq))
dfHA$Isolate_Name <- gsub("\\|(HA).*","",dfHA$Isolate_Name)
dfHA$HAseq <- paste(HAseq)

NPseq <- readDNAStringSet("H3N2_NP_seq.fasta")
length(NPseq)

## [1] 14157

dfNP <- NULL
dfNP$Isolate_Name <- as.character(names(NPseq))
dfNP$Isolate_Name <- gsub("\\|(NP).*","",dfNP$Isolate_Name)
```

```
dfNP$NPseq <- paste(NPseq)

NAseq <- readDNAStringSet("H3N2_NA_seq.fasta")
length(NAseq)

## [1] 14157

dfNA <- NULL
dfNA$Isolate_Name <- as.character(names(NAseq))
dfNA$Isolate_Name <- gsub("\\|(NA).*","",dfNA$Isolate_Name)
dfNA$NAseq <- paste(NAseq)

MPseq <- readDNAStringSet("H3N2_MP_seq.fasta")
length(MPseq)

## [1] 14157

dfMP <- NULL
dfMP$Isolate_Name <- as.character(names(MPseq))
dfMP$Isolate_Name <- gsub("\\|(MP).*","",dfMP$Isolate_Name)
dfMP$MPseq <- paste(MPseq)

NSseq <- readDNAStringSet("H3N2_NS_seq.fasta")
length(NSseq)

## [1] 14157

dfNS <- NULL
dfNS$Isolate_Name <- as.character(names(NSseq))
dfNS$Isolate_Name <- gsub("\\|(NS).*","",dfNS$Isolate_Name)
dfNS$NSseq <- paste(NSseq)

dfseq1 <- unique((merge(merge(dfPB2, dfPB1, by= "Isolate_Name"), dfPA, by=
"Isolate_Name")))
dfseq2 <- unique((merge(merge(dfseq1, dfHA, by= "Isolate_Name"), dfNP, by=
"Isolate_Name")))
dfseq3 <- unique((merge(merge(merge(dfseq2, dfNA, by= "Isolate_Name"), dfMP,
by= "Isolate_Name"),dfNS, by= "Isolate_Name") ))
dim(dfseq3)

## [1] 14157     9
```

Match metadata and sequences

```
dfseq <- merge(dfseq3,metadata,by.x=("Isolate_Name"),by.y=("ID"))

dfseqphylox <- merge(dfseq3,metadataphylox,by.x=("Isolate_Name"),by.y=("ID"))
dfseq3C2a3 <- merge(dfseq3,metadata3C2a3,by.x=("Isolate_Name"),by.y=("ID"))
```

Mutations

Sampling Period

PB2_V255I, PB2_V480I, PB1_G216S, PB1_I517V, HA_S144K, HA_N171K, HA_I406V, HA_G484E, NP_G450S, NP_T472A, NA_G93D, NA_P468L, NA_P468H, NS1_S99T, NS1_L146S

```
NucMut <- data.frame(dfseq$Isolate_Name)
#AA Mutations: Sex
##PB2 V255I -> 763 GTT -> ATT
NucMut$PB2V255I <- str_sub(dfseq$PB2seq,763,763)
NucMut$PB2V255I[NucMut$PB2V255I == "G"] <- 0
NucMut$PB2V255I[NucMut$PB2V255I == "A"] <- 1
NucMut$PB2V255I[NucMut$PB2V255I == "T"] <- NA
NucMut$PB2V255I[NucMut$PB2V255I == "C"] <- NA

#PB1_G216S  -> 646 GGC -> AGC
NucMut$PB1G216S <- (str_sub(dfseq$PB1seq,646,646))
NucMut$PB1G216S[NucMut$PB1G216S == "A"] <- 1
NucMut$PB1G216S[NucMut$PB1G216S == "G"] <- 0
NucMut$PB1G216S[NucMut$PB1G216S == "T"] <- NA
NucMut$PB1G216S[NucMut$PB1G216S == "C"] <- NA

##PB1 I517V -> 1549 ATA -> GTA
NucMut$PB1I517V <- (str_sub(dfseq$PB1seq,1549,1549))
NucMut$PB1I517V[NucMut$PB1I517V == "A"] <- 0
NucMut$PB1I517V[NucMut$PB1I517V == "G"] <- 1
NucMut$PB1I517V[NucMut$PB1I517V == "T"] <- NA
NucMut$PB1I517V[NucMut$PB1I517V == "C"] <- NA

##HA S144K -> 479+480 AGT -> AAA
NucMut$HAS144K <- (str_sub(dfseq$HAseq,479,480))
NucMut$HAS144K[NucMut$HAS144K == "GT"] <- 0
NucMut$HAS144K[NucMut$HAS144K == "AA"] <- 1
NucMut$HAS144K[NucMut$HAS144K == "GG"|NucMut$HAS144K == "TG"|NucMut$HAS144K
== "AG"|NucMut$HAS144K == "CG"|NucMut$HAS144K == "TT"|NucMut$HAS144K ==
"AT"|NucMut$HAS144K == "CT"|NucMut$HAS144K == "GA"|NucMut$HAS144K ==
"TA"|NucMut$HAS144K == "CA"|NucMut$HAS144K == "GC"|NucMut$HAS144K ==
"TC"|NucMut$HAS144K == "AC"|NucMut$HAS144K == "CC"] <- NA

#NA_G93D -> 278 GGC -> GAC
NucMut$NAG93D <- (str_sub(dfseq$NAseq,278,278))
NucMut$NAG93D[NucMut$NAG93D == "A"] <- 1
NucMut$NAG93D[NucMut$NAG93D == "G"] <- 0
NucMut$NAG93D[NucMut$NAG93D == "T"] <- NA
NucMut$NAG93D[NucMut$NAG93D == "C"] <- NA

#NA_P468L -> 1403 CCT -> CTT
NucMut$NAP468L <- (str_sub(dfseq$NAseq,1403,1403))
```

```
NucMut$NAP468L[NucMut$NAP468L == "A"] <- NA
NucMut$NAP468L[NucMut$NAP468L == "G"] <- NA
NucMut$NAP468L[NucMut$NAP468L == "T"] <- 1
NucMut$NAP468L[NucMut$NAP468L == "C"] <- 0

#NA_P468H -> 1403 CCT -> CAT
NucMut$NAP468H <- (str_sub(dfseq$NAseq,1403,1403))
NucMut$NAP468H[NucMut$NAP468H == "A"] <- 1
NucMut$NAP468H[NucMut$NAP468H == "G"] <- NA
NucMut$NAP468H[NucMut$NAP468H == "T"] <- NA
NucMut$NAP468H[NucMut$NAP468H == "C"] <- 0

##NS1 S99T -> 295 TCA -> ACA
NucMut$NS1S99T <- (str_sub(dfseq$NSseq,295,295))
NucMut$NS1S99T[NucMut$NS1S99T == "T"] <- 0
NucMut$NS1S99T[NucMut$NS1S99T == "A"] <- 1
NucMut$NS1S99T[NucMut$NS1S99T == "G"] <- NA
NucMut$NS1S99T[NucMut$NS1S99T == "C"] <- NA

##NS1 L146S -> 437 TTA -> TCA
NucMut$NS1L146S <- (str_sub(dfseq$NSseq,437,437))
NucMut$NS1L146S[NucMut$NS1L146S == "T"] <- 0
NucMut$NS1L146S[NucMut$NS1L146S == "C"] <- 1
NucMut$NS1L146S[NucMut$NS1L146S == "G"] <- NA
NucMut$NS1L146S[NucMut$NS1L146S == "A"] <- NA

NucMut[] <- lapply(NucMut,factor)
row.names(NucMut) <- NucMut$dfseq.Isolate_Name

NucMutPeriod <- data.frame(row.names(NucMut), NucMut$PB2V255I,
NucMut$PB1G216S, NucMut$PB1I517V, NucMut$HAS144K, NucMut$NAG93D,
NucMut$NAP468L, NucMut$NAP468H, NucMut$NS1S99T, NucMut$NS1L146S)

NucMutPeriod <- NucMut %>%
  select(PB2V255I, PB1G216S, PB1I517V, HAS144K, NAG93D, NAP468L, NAP468H,
NS1S99T, NS1L146S)

NucMutPeriod$Isolate_Name <- row.names(NucMutPeriod)
```

Mutations: Sex

```
NucMut <- data.frame(dfseq$Isolate_Name)
#AA Mutations: Sex
##PB2 V255I -> 763 GTT -> ATT
NucMut$PB2V255I <- str_sub(dfseq$PB2seq,763,763)
NucMut$PB2V255I[NucMut$PB2V255I == "G"] <- 0
NucMut$PB2V255I[NucMut$PB2V255I == "A"] <- 1
NucMut$PB2V255I[NucMut$PB2V255I == "T"] <- NA
NucMut$PB2V255I[NucMut$PB2V255I == "C"] <- NA
```

```
##PB2 V480I -> 1438 GTC -> ATC
NucMut$PB2V480I <- (str_sub(dfseq$PB2seq,1438,1438))
NucMut$PB2V480I[NucMut$PB2V480I == "G"] <- 0
NucMut$PB2V480I[NucMut$PB2V480I == "A"] <- 1
NucMut$PB2V480I[NucMut$PB2V480I == "T"] <- NA
NucMut$PB2V480I[NucMut$PB2V480I == "C"] <- NA

##HA S144K -> 479+480 AGT -> AAA
NucMut$HAS144K <- (str_sub(dfseq$HAseq,479,480))
NucMut$HAS144K[NucMut$HAS144K == "GT"] <- 0
NucMut$HAS144K[NucMut$HAS144K == "AA"] <- 1
NucMut$HAS144K[NucMut$HAS144K == "GG"|NucMut$HAS144K == "TG"|NucMut$HAS144K
== "AG"|NucMut$HAS144K == "CG"|NucMut$HAS144K == "TT"|NucMut$HAS144K ==
"AT"|NucMut$HAS144K == "CT"|NucMut$HAS144K == "GA"|NucMut$HAS144K ==
"TA"|NucMut$HAS144K == "CA"|NucMut$HAS144K == "GC"|NucMut$HAS144K ==
"TC"|NucMut$HAS144K == "AC"|NucMut$HAS144K == "CC"] <- NA

##HA N171K -> 561 AAT -> AAG
NucMut$HAN171K <- (str_sub(dfseq$HAseq,561,561))
NucMut$HAN171K[NucMut$HAN171K == "T"] <- 0
NucMut$HAN171K[NucMut$HAN171K == "G"] <- 1
NucMut$HAN171K[NucMut$HAN171K == "A"] <- NA
NucMut$HAN171K[NucMut$HAN171K == "C"] <- NA

##HA I406V -> 1264 ATT -> GTT
NucMut$HAI406V <- (str_sub(dfseq$HAseq,1264,1264))
NucMut$HAI406V[NucMut$HAI406V == "A"] <- 0
NucMut$HAI406V[NucMut$HAI406V == "G"] <- 1
NucMut$HAI406V[NucMut$HAI406V == "T"] <- NA
NucMut$HAI406V[NucMut$HAI406V == "C"] <- NA

##HA_G484E -> 1499 GGA -> GAA
NucMut$HAG484E <- (str_sub(dfseq$HAseq,1499,1499))
NucMut$HAG484E[NucMut$HAG484E == "G"] <- 0
NucMut$HAG484E[NucMut$HAG484E == "A"] <- 1
NucMut$HAG484E[NucMut$HAG484E == "T"] <- NA
NucMut$HAG484E[NucMut$HAG484E == "C"] <- NA

##NP G450S -> 1348 GGT -> AGT
NucMut$NPG450S <- (str_sub(dfseq$NPseq,1348,1348))
NucMut$NPG450S[NucMut$NPG450S == "G"] <- 0
NucMut$NPG450S[NucMut$NPG450S == "A"] <- 1
NucMut$NPG450S[NucMut$NPG450S == "T"] <- NA
NucMut$NPG450S[NucMut$NPG450S == "C"] <- NA

##NP T472A -> 1414 ACG -> GCG
NucMut$NPT472A <- (str_sub(dfseq$NPseq,1414,1414))
NucMut$NPT472A[NucMut$NPT472A == "A"] <- 0
```

```
NucMut$NPT472A[NucMut$NPT472A == "G"] <- 1
NucMut$NPT472A[NucMut$NPT472A == "T"] <- NA
NucMut$NPT472A[NucMut$NPT472A == "C"] <- NA

##NS1 S99T -> 295 TCA -> ACA
NucMut$NS1S99T <- (str_sub(dfseq$NSseq,295,295))
NucMut$NS1S99T[NucMut$NS1S99T == "T"] <- 0
NucMut$NS1S99T[NucMut$NS1S99T == "A"] <- 1
NucMut$NS1S99T[NucMut$NS1S99T == "G"] <- NA
NucMut$NS1S99T[NucMut$NS1S99T == "C"] <- NA

##NS1 L146S -> 437 TTA -> TCA
NucMut$NS1L146S <- (str_sub(dfseq$NSseq,437,437))
NucMut$NS1L146S[NucMut$NS1L146S == "T"] <- 0
NucMut$NS1L146S[NucMut$NS1L146S == "C"] <- 1
NucMut$NS1L146S[NucMut$NS1L146S == "G"] <- NA
NucMut$NS1L146S[NucMut$NS1L146S == "A"] <- NA

NucMut[] <- lapply(NucMut,factor)
row.names(NucMut) <- NucMut$dfseq.Isolate_Name
NucMutSex <- data.frame(row.names(NucMut), NucMut$PB2V255I, NucMut$PB2V480I,
NucMut$HAS144K, NucMut$HAN171K, NucMut$HAI406V, NucMut$HAG484E,
NucMut$NPG450S, NucMut$NPT472A, NucMut$NS1S99T, NucMut$NS1L146S)

NucMutSex <- NucMut %>%
  select(PB2V255I, PB2V480I, HAS144K, HAN171K, HAI406V, HAG484E, NPG450S,
NPT472A, NS1S99T, NS1L146S)
NucMutSex$Isolate_Name <- row.names(NucMutSex)
```

Boxplot

Sampling Period

```
metadataPeriod <-
merge(NucMutPeriod,metadata,by.x=("Isolate_Name"),by.y=("ID"))
metadataPeriod$Collection_Data <-
as.Date(as.character(metadataPeriod$Collection_Data), "%Y-%m-%d")

metadataPeriodphylox <-
merge(NucMutPeriod,metadataphylox,by.x=("Isolate_Name"),by.y=("ID"))
metadataPeriodphylox$Collection_Data <-
as.Date(as.character(metadataPeriodphylox$Collection_Data), "%Y-%m-%d")

metadataPeriod3c2a <-
merge(NucMutPeriod,metadata3C2a,by.x=("Isolate_Name"),by.y=("ID"))
metadataPeriod3c2a$Collection_Data <-
as.Date(as.character(metadataPeriod3c2a$Collection_Data), "%Y-%m-%d")

metadataPeriod3C2a3 <-
merge(NucMutPeriod,metadata3C2a3,by.x=("Isolate_Name"),by.y=("ID"))
```

```
metadataPeriod3C2a3$Collection_Data <-
as.Date(as.character(metadataPeriod3C2a3$Collection_Data), "%Y-%m-%d")

analyze <- function(filename,mutation) {


  Period_array <- NULL

    Period1 <- subset(filename, Collection_Data >= as.Date("2016-04-15") &
Collection_Data <= as.Date("2016-11-05"))
      Period1$Period <- "0"

    Period2 <- subset(filename, Collection_Data >= as.Date("2016-11-05") &
Collection_Data <= as.Date("2017-04-17"))
    Period2$Period <- "1"

    Period3 <- subset(filename, Collection_Data >= as.Date("2017-04-17") &
Collection_Data <= as.Date("2017-10-01"))
    Period3$Period <- "2"

    filePeriod <- rbind(Period1,Period2, Period3)
    #Between these period NA P468 is not occuring, so everything would be 1
    filePeriod$NAP468L[is.na(filePeriod$NAP468L)] <-0
    filePeriod$NAP468H[is.na(filePeriod$NAP468H)] <-0

    mutation <- deparse(substitute(mutation))

    NucMutPeriod2 <-
cbind.data.frame(Isolate_Name=filePeriod$Isolate_Name,mutation=as.factor(file
Period[,mutation]),Period=filePeriod$Period)
    NucMutPeriod2 <- NucMutPeriod2[!is.na(NucMutPeriod2$mutation),]
    NucMutPeriod2 <- NucMutPeriod2[!is.na(NucMutPeriod2$Period),]


    sum_Period1_subset <- (subset(NucMutPeriod2, Period == "0", select =
mutation) %>% unlist)
    sum_Period2_subset <- (subset(NucMutPeriod2, Period == "1", select =
mutation) %>% unlist)
    sum_Period3_subset <- (subset(NucMutPeriod2, Period == "2", select =
mutation) %>% unlist)

    len <-
min(c(length(sum_Period1_subset),length(sum_Period2_subset),length(sum_Period
3_subset)))*0.95


    box_Period1 <-
      replicate(1000,{AllPeriod1asymp <- sample(sum_Period1_subset,len)
      length(which(AllPeriod1asymp==1))})
```

```
    box_Period2 <-
      replicate(1000,{AllPeriod2asymp <- sample(sum_Period2_subset,len)
      length(which(AllPeriod2asymp==1))})


    box_Period3 <-
      replicate(1000,{AllPeriod3asymp <- sample(sum_Period3_subset,len)
      length(which(AllPeriod3asymp==1))})

    Period_array$Period1<- (box_Period1)
    Period_array$Period2<- (box_Period2)
    Period_array$Period3<- (box_Period3)
    Period_array <- as.data.frame(Period_array)
    Period_array <- reshape2::melt(Period_array)
    colnames(Period_array) <- c("Period","Mutations")

    Period_array$Period <- as.character(Period_array$Period)
    Period_array$Period[(Period_array$Period) == "Period1"] <-
paste0("Period1 (",length(which(sum_Period1_subset==1)),")")
Period_array$Period[(Period_array$Period) == "Period2"] <- paste0("Period2
(",length(which(sum_Period2_subset==1)),")")
Period_array$Period[(Period_array$Period) == "Period3"] <- paste0("Period3
(",length(which(sum_Period3_subset==1)),")")


    ylab_name <-
paste(substitute(filename),"_",substitute(mutation),")",sep="")
    image_name <- paste(substitute(filename),
"_Boxplot_Period_",substitute(mutation),".png",sep="")

    scale_value <- 0.5

boxtotalPeriod <- ggplot(Period_array, aes(factor(Period), Mutations),
fill=as.factor(Period)) + geom_boxplot(position =
position_dodge(width=100),outlier.size = 0.05*scale_value)
boxtotalPeriod <- boxtotalPeriod + xlab("Period") +  ylab(ylab_name) +
guides(fill=guide_legend(title="Period"))

boxtotalPeriod <- boxtotalPeriod + expand_limits(x=0,y=0)
boxtotalPeriod <- boxtotalPeriod + geom_point(aes(y=Mutations, group=Period),
position = "jitter", size = 0.0005*scale_value)
boxtotalPeriod <- boxtotalPeriod + theme(plot.background = element_rect(fill
= "transparent", color = NA),text = element_text(size = 12*scale_value),
axis.title.x = element_blank()) # bg of the plot
boxtotalPeriod

ggsave(image_name, plot = boxtotalPeriod, device = NULL, path =
```

```
"//sciensano.be/fs/1540_TAG_Employee/6_R&D/PROJECTS/On-going/Be READY_Viro/5.
Research/Surveillance samples H3N2/2. mutations/Statistics/GISAID
Check/Permutation test", width = 10*scale_value, height
=8*scale_value,units="cm", dpi = 150)


if(median(box_Period2) > median(box_Period3)) {
    pPeriodComboPeak_End <- 0.975*(median(box_Period2) - median(box_Period3))
    pPeriodComboPeak_End1 <- median(box_Period2)+pPeriodComboPeak_End
    pPeriodComboPeak_End2 <- median(box_Period2)-pPeriodComboPeak_End
    spPeriodComboPeak_End <-
sum(length(which(box_Period2>pPeriodComboPeak_End1)))+sum(length(which(box_Pe
riod2<pPeriodComboPeak_End2)))
    a <- paste0("P-value (Period: Peak vs End) =
",spPeriodComboPeak_End/1000)
} else {
  pPeriodComboPeak_End <- 0.975*(median(box_Period3) - median(box_Period2))
    pPeriodComboPeak_End1 <- median(box_Period3)+pPeriodComboPeak_End
    pPeriodComboPeak_End2 <- median(box_Period3)-pPeriodComboPeak_End
    spPeriodComboPeak_End <-
sum(length(which(box_Period3>pPeriodComboPeak_End1)))+sum(length(which(box_Pe
riod3<pPeriodComboPeak_End2)))
    a <- paste0("P-value (Period: Peak vs End) =
",spPeriodComboPeak_End/1000)
}

if(median(box_Period1) > median(box_Period3)) {
    pPeriodComboBeginning_End <- 0.975*(median(box_Period1) -
median(box_Period3))
    pPeriodComboBeginning_End1 <-
median(box_Period1)+pPeriodComboBeginning_End
    pPeriodComboBeginning_End2 <- median(box_Period1)-
pPeriodComboBeginning_End
    spPeriodComboBeginning_End <-
sum(length(which(box_Period1>pPeriodComboBeginning_End1)))+sum(length(which(b
ox_Period1<pPeriodComboBeginning_End2)))
    b <- paste0("P-value (Period: Beginning vs End) =
",spPeriodComboBeginning_End/1000)
} else {
    pPeriodComboBeginning_End <- 0.975*(median(box_Period3) -
median(box_Period1))
    pPeriodComboBeginning_End1 <-
median(box_Period3)+pPeriodComboBeginning_End
    pPeriodComboBeginning_End2 <- median(box_Period3)-
pPeriodComboBeginning_End
    spPeriodComboBeginning_End <-
sum(length(which(box_Period3>pPeriodComboBeginning_End1)))+sum(length(which(b
ox_Period3<pPeriodComboBeginning_End2)))
    b <- paste0("P-value (Period: Beginning vs End) =
",spPeriodComboBeginning_End/1000)
```

```
}

if(median(box_Period1) > median(box_Period2)) {
    pPeriodComboBeginning_Peak <- 0.975*(median(box_Period1) - 
median(box_Period2))
    pPeriodComboBeginning_Peak1 <- 
median(box_Period1)+pPeriodComboBeginning_Peak
    pPeriodComboBeginning_Peak2 <- median(box_Period1)-
pPeriodComboBeginning_Peak
    spPeriodComboBeginning_Peak <- 
sum(length(which(box_Period1>pPeriodComboBeginning_Peak1)))+sum(length(which(
box_Period1<pPeriodComboBeginning_Peak2)))
    c <- paste0("P-value (Period: Beginning vs Peak) = 
",spPeriodComboBeginning_End/1000)
} else {
  pPeriodComboBeginning_Peak <- 0.975*(median(box_Period2) - 
median(box_Period1))
    pPeriodComboBeginning_Peak1 <- 
median(box_Period2)+pPeriodComboBeginning_Peak
    pPeriodComboBeginning_Peak2 <- median(box_Period2)-
pPeriodComboBeginning_Peak
    spPeriodComboBeginning_Peak <- 
sum(length(which(box_Period2>pPeriodComboBeginning_Peak1)))+sum(length(which(
box_Period2<pPeriodComboBeginning_Peak2)))
    c <- paste0("P-value (Period: Beginning vs Peak) = 
",spPeriodComboBeginning_Peak/1000)
}


df <- c(a,b,c)
df <- as.data.frame(df)
df <- separate(data = df, col = df, into = c("left", "right"), sep = "=")
dfname <- paste0("//sciensano.be/fs/1540_TAG_Employee/6_R&D/PROJECTS/On-
going/Be READY_Viro/5. Research/Surveillance samples H3N2/2. 
mutations/Statistics/GISAID Check/Permutation test/",substitute(filename),
"_Boxplot_Period_",substitute(mutation),".csv")
write.csv(df, file = dfname)
}

metadatalist <- c("metadataPeriod", 
"metadataPeriodphylox","metadataPeriod3c2a","metadataPeriod3C2a3")
mutationlist <- c("PB2V255I", "PB1G216S","PB1I517V", "HAS144K" ,"NAG93D"
,"NAP468L","NAP468H", "NS1S99T", "NS1L146S")
analyzelist <- outer(metadatalist,mutationlist,paste, sep=",")
analyzelist <- paste("analyze(", analyzelist,")", sep="")

lapply(analyzelist, function(x) {eval(parse(text = x))})
```

Sex

```
metadataSex <- merge(NucMutSex,metadata,by.x=("Isolate_Name"),by.y=("ID"))
metadataSex$Collection_Data <-
as.Date(as.character(metadataSex$Collection_Data), "%Y-%m-%d")
metadataSex <- subset(metadataSex, Collection_Data >= as.Date("2016-04-15") &
Collection_Data <= as.Date("2017-10-01"))

metadataSexphylox <-
merge(NucMutSex,metadataphylox,by.x=("Isolate_Name"),by.y=("ID"))
metadataSexphylox$Collection_Data <-
as.Date(as.character(metadataSexphylox$Collection_Data), "%Y-%m-%d")
metadataSexphylox <- subset(metadataSexphylox, Collection_Data >=
as.Date("2016-04-15") & Collection_Data <= as.Date("2017-10-01"))

metadataSex3c2a <-
merge(NucMutSex,metadata3C2a,by.x=("Isolate_Name"),by.y=("ID"))
metadataSex3c2a$Collection_Data <-
as.Date(as.character(metadataSex3c2a$Collection_Data), "%Y-%m-%d")
metadataSex3c2a <- subset(metadataSex3c2a, Collection_Data >= as.Date("2016-
04-15") & Collection_Data <= as.Date("2017-10-01"))

metadataSex3C2a3 <-
merge(NucMutSex,metadata3C2a3,by.x=("Isolate_Name"),by.y=("ID"))
metadataSex3C2a3$Collection_Data <-
as.Date(as.character(metadataSex3C2a3$Collection_Data), "%Y-%m-%d")
metadataSex3C2a3 <- subset(metadataSex3C2a3, Collection_Data >=
as.Date("2016-04-15") & Collection_Data <= as.Date("2017-10-01"))

analyze <- function(filename,mutation) {
    Sex_array <- NULL
    mutation <- deparse(substitute(mutation))

    NucMutSex1 <-
cbind.data.frame(Isolate_Name=filename$Isolate_Name,mutation=as.factor(filena
me[,mutation]),Sex=filename$Host_gender)
    NucMutSex1 <- NucMutSex1[!is.na(NucMutSex1$mutation),]
    NucMutSex1 <- NucMutSex1[!is.na(NucMutSex1$Sex),]

    sum_Male_subset <- (subset(NucMutSex1, Sex == "Male", select = mutation)
%>% unlist)
    sum_Female_subset <- (subset(NucMutSex1, Sex == "Female", select =
mutation) %>% unlist)


    len <- min(c(length(sum_Male_subset),length(sum_Female_subset)))


    box_Male <-
      replicate(1000,{AllMaleasymp <- sample(sum_Male_subset,len)
```

```
      length(which(AllMaleasymp==1))})


    box_Female <-
      replicate(1000,{AllFemaleasymp <- sample(sum_Female_subset,len)
      length(which(AllFemaleasymp==1))})

    Sex_array$Male<- (box_Male)
    Sex_array$Female<- (box_Female)
    Sex_array <- as.data.frame(Sex_array)
    Sex_array <- reshape2::melt(Sex_array)
    colnames(Sex_array) <- c("Sex","Mutations")

    Sex_array$Sex <- as.character(Sex_array$Sex)
    Sex_array$Sex[(Sex_array$Sex) == "Male"] <- paste0("Male
(",length(which(sum_Male_subset==1)),")")
    Sex_array$Sex[(Sex_array$Sex) == "Female"] <- paste0("Female
(",length(which(sum_Female_subset==1)),")")

    ylab_name <-
paste(substitute(filename),"_",substitute(mutation),")",sep="")
    image_name <- paste(substitute(filename),
"_Boxplot_Sex_",substitute(mutation),".png",sep="")


    scale_value <- 0.35

    boxtotalSex <- ggplot(Sex_array, aes(factor(Sex), Mutations),
fill=as.factor(Sex)) + geom_boxplot(position = position_dodge(width=100),size
= 0.05*scale_value)
    boxtotalSex <- boxtotalSex + xlab("Sex") +  ylab(ylab_name) +
guides(fill=guide_legend(title="Sex"))
    boxtotalSex <- boxtotalSex + geom_point(aes(y=Mutations, group=Sex),
position = "jitter", size = 0.0005*scale_value)
    boxtotalSex <- boxtotalSex + theme(plot.background = element_rect(fill =
"transparent", color = NA),text = element_text(size = 12*scale_value),
axis.title.x = element_blank()) # bg of the plot
    boxtotalSex

    ggsave(image_name, plot = boxtotalSex, device = NULL, path =
"//sciensano.be/fs/1540_TAG_Employee/6_R&D/PROJECTS/On-going/Be READY_Viro/5.
Research/Surveillance samples H3N2/2. mutations/Statistics/GISAID
Check/Permutation test", width = 10*scale_value, height
=8*scale_value,units="cm", dpi = 150)


      if (median(box_Female) > median(box_Male)){
      psurvCombo <- 0.975*((median(box_Female) - median(box_Male)))
      psurvCombo1 <- median(box_Female)+psurvCombo
```

```
      psurvCombo2 <- median(box_Female)-psurvCombo
      spsurvCombo <-
sum(length(which(box_Female>psurvCombo1)))+sum(length(which(box_Female<psurvC
ombo2)))
      a <- paste0("P-value (Sex) =",spsurvCombo/1000)

      df <- c(a)
      df <- as.data.frame(df)
      df <- separate(data = df, col = df, into = c("left", "right"), sep =
"=")
  } else {
      psurvCombo <- 0.975*((median(box_Male) - median(box_Female)))
      psurvCombo1 <- median(box_Male)+psurvCombo
      psurvCombo2 <- median(box_Male)-psurvCombo
      spsurvCombo <-
sum(length(which(box_Male>psurvCombo1)))+sum(length(which(box_Male<psurvCombo
2)))
      a <- paste0("P-value (Sex) =",spsurvCombo/1000)

      df <- c(a)
      df <- as.data.frame(df)
      df <- separate(data = df, col = df, into = c("left", "right"), sep =
"=")
  }

    dfname <- paste0("//sciensano.be/fs/1540_TAG_Employee/6_R&D/PROJECTS/On-
going/Be READY_Viro/5. Research/Surveillance samples H3N2/2.
mutations/Statistics/GISAID Check/Permutation test/",substitute(filename),
"_Boxplot_Sex_",substitute(mutation),".csv")
    write.csv(df, file = dfname)


}
metadatalist <- c("metadataSex",
"metadataSexphylox","metadataSex3c2a","metadataSex3C2a3")
mutationlist <- c("PB2V255I", "PB2V480I", "HAS144K" ,"HAN171K"
,"HAI406V","HAG484E", "NPG450S", "NPT472A", "NS1S99T", "NS1L146S")
analyzelist <- outer(metadatalist,mutationlist,paste, sep=",")
analyzelist <- paste("analyze(", analyzelist,")", sep="")

lapply(analyzelist, function(x) {eval(parse(text = x))})
```

Proportion fatalities in renal insufficiency patients

There are 7/35 patients with renal insufficiency that died, while in the total dataset this is 19/253.

```
renal <- c(7,28)

chisq.test(renal, p = c(19/253,234/253))

#The p-value is 0.005051, which is less than alpha=0.05. We can conclude that
the colors are significantly not commonly distributed with a p-value of
0.005051. The observed proportion is significantly different from the
expected proportions
```
