# Supplementary material for: Whole-Genome Sequence Approach and Phylogenomic Stratification Improve the Association Analysis of Mutations With Patient Data in Influenza Surveillance
Source: Front Microbiol. 2022 Apr 19;13:809887. doi: 10.3389/fmicb.2022.809887 (PMC9063638; doi:10.3389/fmicb.2022.809887)
Supplement: Supplementary file 2 [file Data_Sheet_2.PDF]

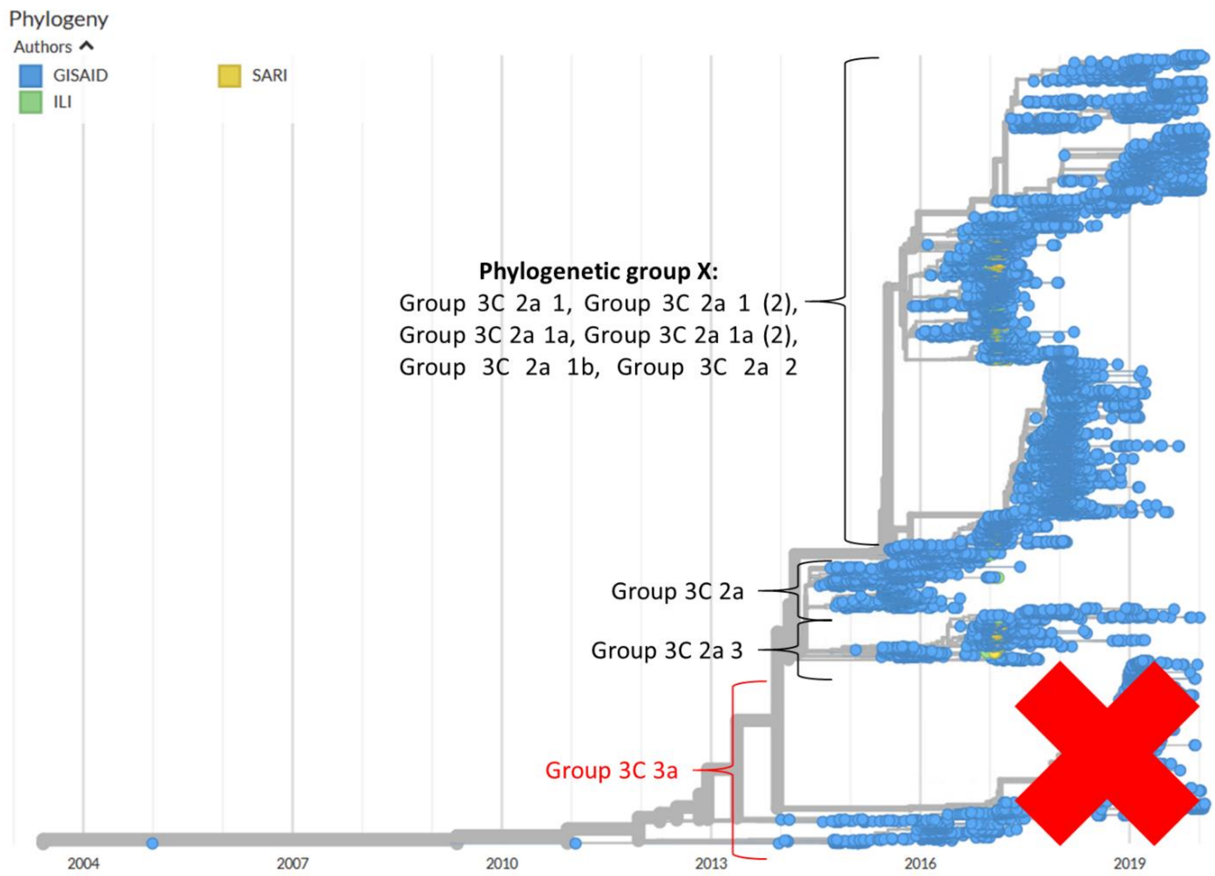

Supplementary Figure S1: Overview of influenza samples from the Belgian 2016-2017 flu season in the context of globally circulating influenza strains based on an in-house build of Nextstrain using only whole genome sequence information. The green and yellow dots represent ILI (mild infections) and SARI (moderate/severe infections) samples sequenced in this study, while the blue dots represent samples selected from the GISAID database. This tree contains 14 178 genomes. To leave out samples that were phylogenetically distant from the Belgian samples, the samples belonging to the Group 3C 3a were omitted.

Supplementary Figure S2: Comparison of the Belgian influenza samples with the GISAID database for mutations that were considered significant related to the sex, but were afterwards discarded because when considering the viral background it changed. Also, comparison of the distribution of the samples in the phylogenetic groups for the significant results after running the Fisher's exact test with FDR correction when taking all samples into consideration. These GISAID samples include samples collected in the same period as in Figure 3. In the graphs representing the situation in Belgian, above the bars the number of samples that had this mutations are indicated. Based on the graphs coming from the Belgian data, we concluded that the male and female patients were unequally distributed in the two subgroups, Group 3C2a3 and Phylogenetic group X. For example for the PB2-V255I mutation there are 34 out of 122 male patients and 17 out of of female patients which is a significant difference. However, all of these samples belong to Group 3C2a3 resulting in 34 out of 40 males and 17 out of 18 males which gives a completely distribution.

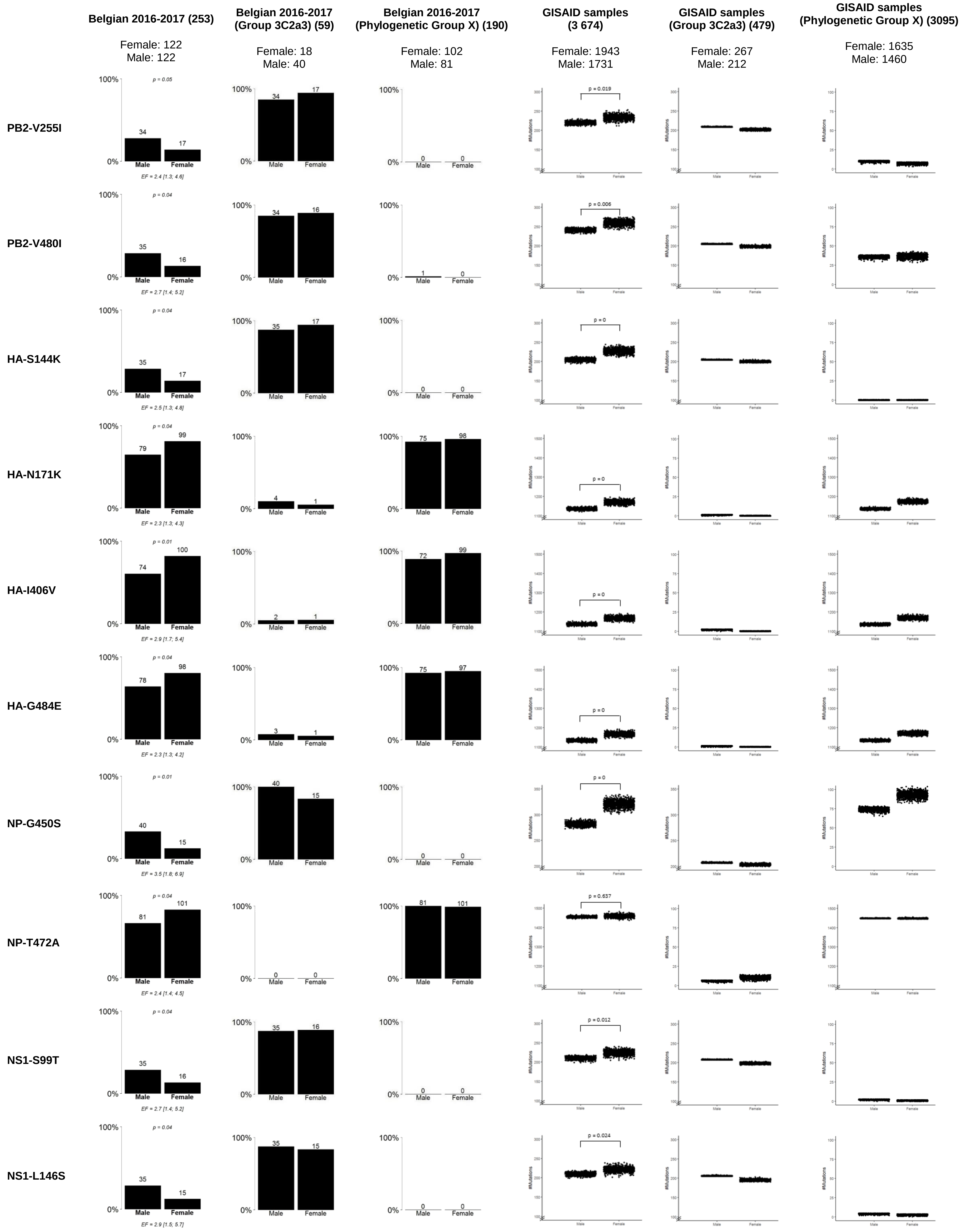

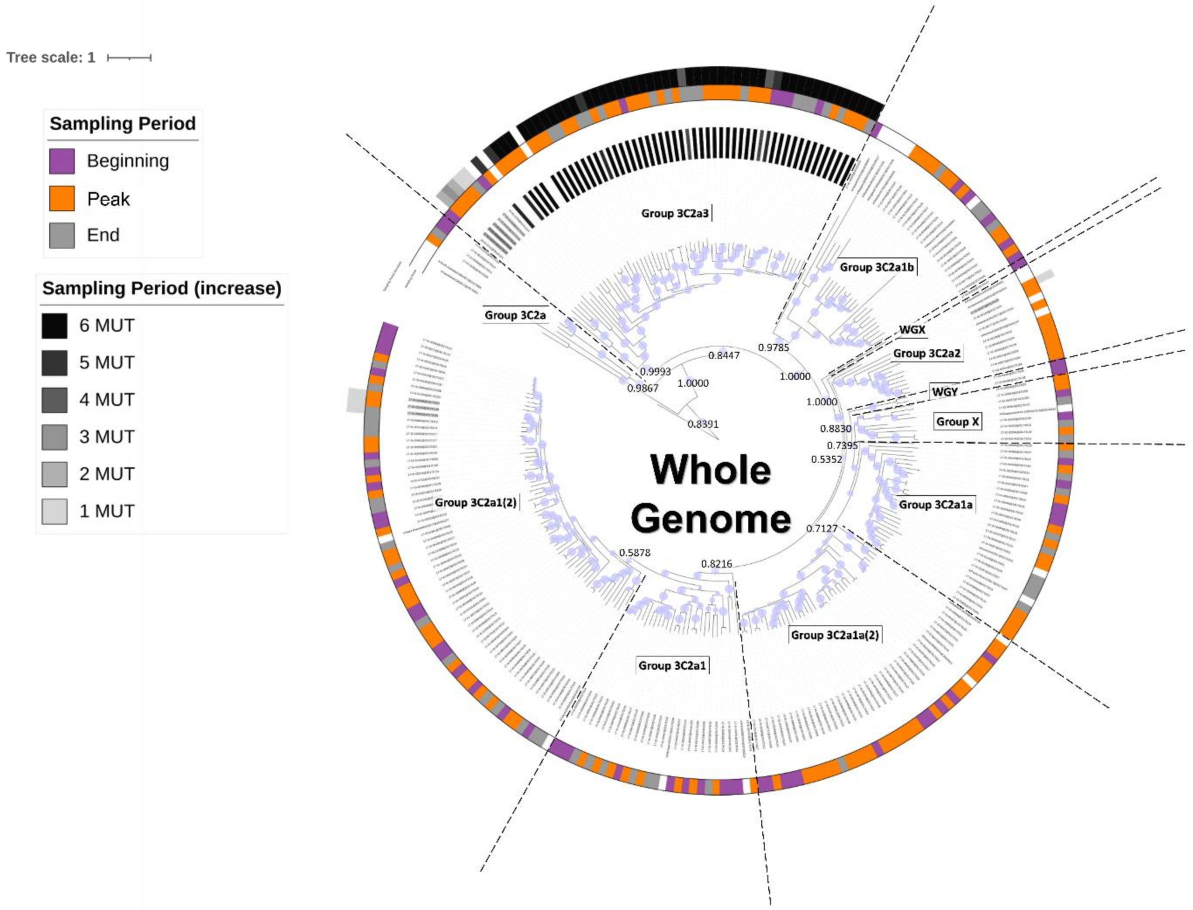

Supplementary Figure S3: Phylogenetic tree based on the whole H3N2 genome annotated with the Sampling Period (emergence over time). The colored rings around the tree represent the Sampling Period (Purple=Beginning; Orange=Peak; Grey=End) and the number of associated mutations (PB2-V255I, HA-S144K, NA-G93D, NA-P468L, NS1-S99T, and/or NS1-L146S) that occur together. Group "X" clustered together in a separate cluster from the other phylogenetic groups. Groups "WGX" and "WGY" contain samples that could not be classified. Groups labelled with the segment name and a single letter (e.g. PB1X) similarly represent any remaining samples that could not be confidently assigned into phylogenetic groups according to their segment trees. Within the tree, the group labels represent the phylogenetic groups that were assigned to their respective samples according to their classification based on references (colored names) and the support of nodes by posterior probability values. Posterior probability values are indicated on key nodes that separate phylogenetic groups. The size of blue disks on nodes represents the posterior probability scaled between 0.5 and 1. The scale bar represents the average number of substitutions per site.

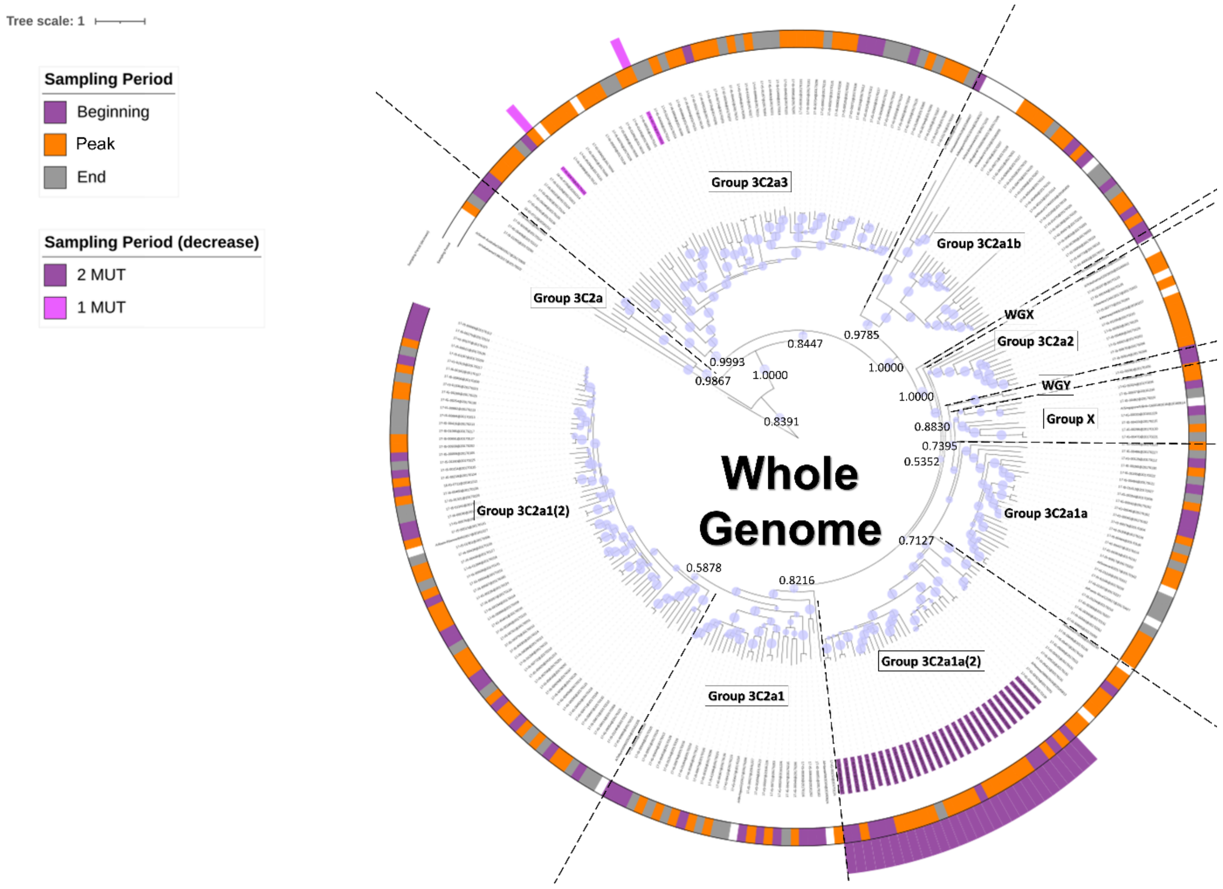

Supplementary Figure S4: Phylogenetic tree based on the whole H3N2 genome annotated with the Sampling Period (decrease over time). The colored rings around the tree represent the Sampling Period (Purple=Beginning; Orange=Peak; Grey=End) and the number of associated mutations (PB1-G216S, and/or PB1-I517V) that occur together. Group "X" clustered together in a separate cluster from the other phylogenetic groups. Groups "WGX" and "WGY" contain samples that could not be classified. Groups labelled with the segment name and a single letter (e.g. PB1X) similarly represent any remaining samples that could not be confidently assigned into phylogenetic groups according to their segment trees. Within the tree, the group labels represent the phylogenetic groups that were assigned to their respective samples according to their classification based on references (colored names) and the support of nodes by posterior probability values. Posterior probability values are indicated on key nodes that separate phylogenetic groups. The size of blue disks on nodes represents the posterior probability scaled between 0.5 and 1. The scale bar represents the average number of substitutions per site.

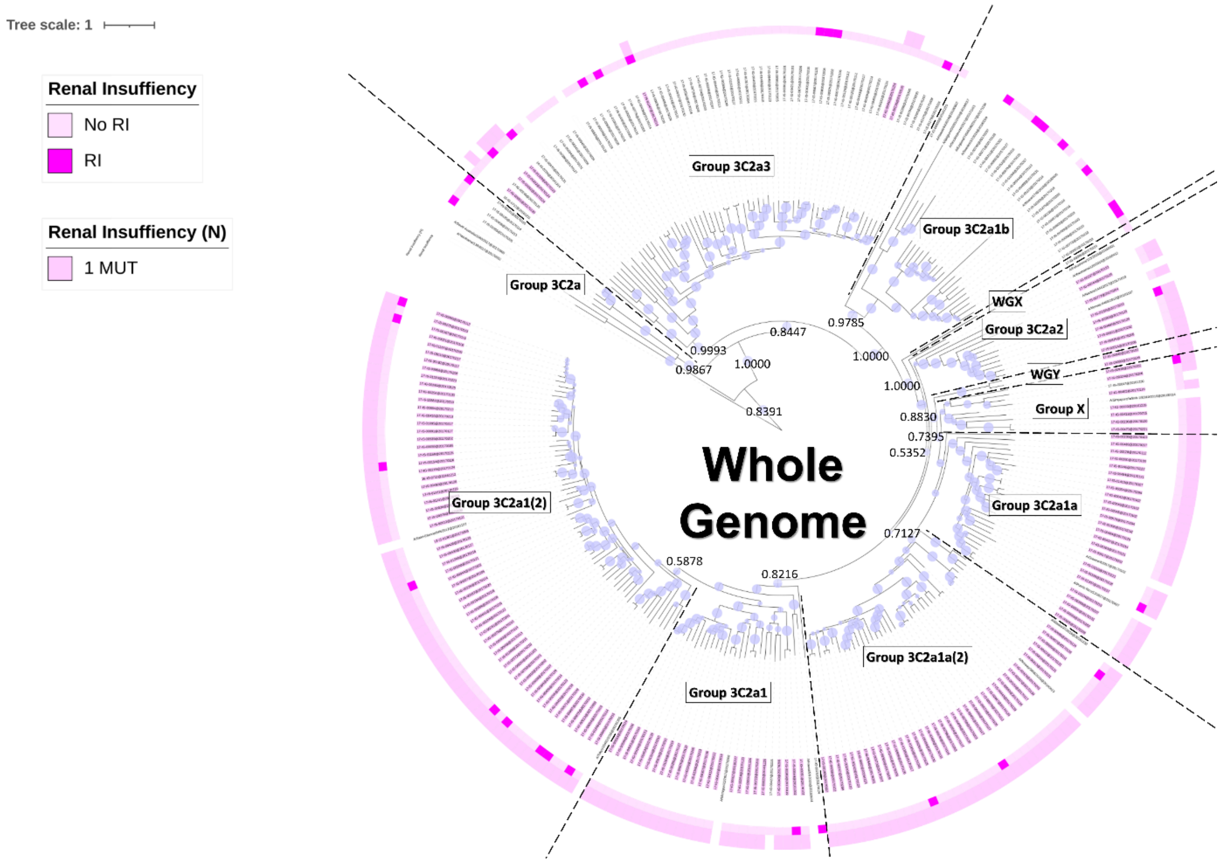

Supplementary Figure S5: Phylogenetic tree based on the whole H3N2 genome annotated with the Renal Insuffiency (No renal Insuffiency). The colored rings around the tree represent the Renal insufficiency (Light pink=No Renal Insufficiency; Dark Pink=Renal Insufficiency) and the number of associated mutations (PB2-R299K) that occur together. Group "X" clustered together in a separate cluster from the other phylogenetic groups. Groups "WGX" and "WGY" contain samples that could not be classified. Groups labelled with the segment name and a single letter (e.g. PB1X) similarly represent any remaining samples that could not be confidently assigned into phylogenetic groups according to their segment trees. Within the tree, the group labels represent the phylogenetic groups that were assigned to their respective samples according to their classification based on references (colored names) and the support of nodes by posterior probability values. Posterior probability values are indicated on key nodes that separate phylogenetic groups. The size of blue disks on nodes represents the posterior probability scaled between 0.5 and 1. The scale bar represents the average number of substitutions per site.

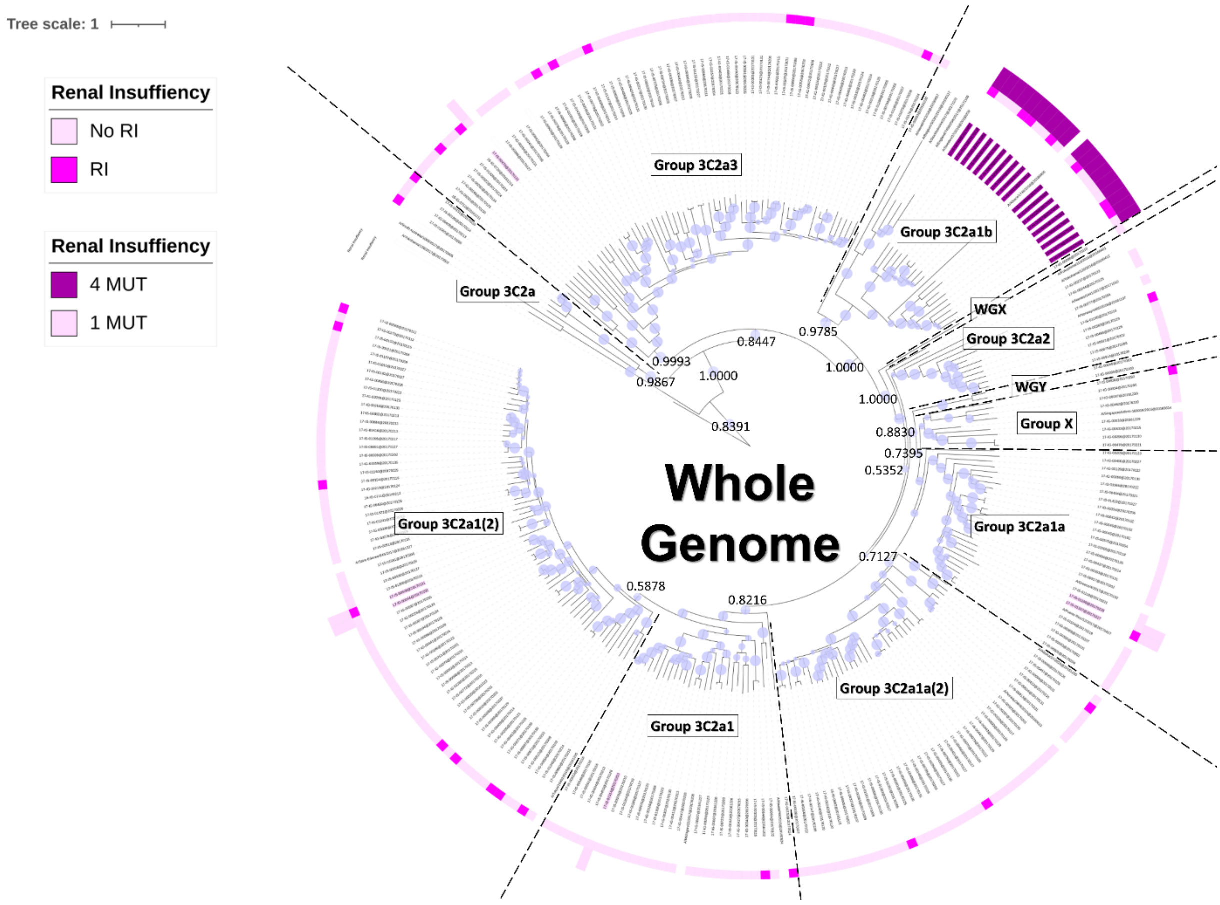

Supplementary Figure S6: Phylogenetic tree based on the whole H3N2 genome annotated with the Renal Insuffiency (Renal Insuffiency). The colored rings around the tree represent the Renal insufficiency (Light pink=No Renal Insufficiency; Dark Pink=Renal Insufficiency) and the number of associated mutations (PB2-K340R, HA-K92R, HA-H311Q, and/or NP-V197I) that occur together. Group "X" clustered together in a separate cluster from the other phylogenetic groups. Groups "WGX" and "WGY" contain samples that could not be classified. Groups labelled with the segment name and a single letter (e.g. PB1X) similarly represent any remaining samples that could not be confidently assigned into phylogenetic groups according to their segment trees. Within the tree, the group labels represent the phylogenetic groups that were assigned to their respective samples according to their classification based on references (colored names) and the support of nodes by posterior probability values. Posterior probability values are indicated on key nodes that separate phylogenetic groups. The size of blue disks on nodes represents the posterior probability scaled between 0.5 and 1. The scale bar represents the average number of substitutions per site.
